# Pupil dilation reflects effortful action invigoration in overcoming aversive Pavlovian biases

**DOI:** 10.3758/s13415-024-01191-y

**Published:** 2024-05-21

**Authors:** Johannes Algermissen, Hanneke E. M. den Ouden

**Affiliations:** 1 Donders Institute for Brain, Radboud University, Cognition, and Behaviour, Thomas van Aquinostraat 4, 6526 GD Nijmegen, The Netherlands; 2 Department of Experimental Psychology, University of Oxford, Oxford, UK

**Keywords:** Motivational biases, Pavlovian, Pupillometry, Eye-tracker, Effort, Invigoration

## Abstract

“Pavlovian” or “motivational” biases describe the phenomenon that the valence of prospective outcomes modulates action invigoration: Reward prospect invigorates action, whereas punishment prospect suppresses it. The adaptive role of these biases in decision-making is still unclear. One idea is that they constitute a fast-and-frugal decision strategy in situations characterized by high arousal, e.g., in presence of a predator, which demand a quick response. In this pre-registered study (*N* = 35), we tested whether such a situation—induced via subliminally presented angry versus neutral faces—leads to increased reliance on Pavlovian biases. We measured trial-by-trial arousal by tracking pupil diameter while participants performed an orthogonalized Motivational Go/NoGo Task. Pavlovian biases were present in responses, reaction times, and even gaze, with lower gaze dispersion under aversive cues reflecting “freezing of gaze.” The subliminally presented faces did not affect responses, reaction times, or pupil diameter, suggesting that the arousal manipulation was ineffective. However, pupil dilations reflected facets of bias suppression, specifically the physical (but not cognitive) effort needed to overcome aversive inhibition: Particularly strong and sustained dilations occurred when participants managed to perform Go responses to aversive cues. Conversely, no such dilations occurred when they managed to inhibit responses to Win cues. These results suggest that pupil diameter does not reflect response conflict per se nor the inhibition of prepotent responses, but specifically effortful action invigoration as needed to overcome aversive inhibition. We discuss our results in the context of the “value of work” theory of striatal dopamine.

**Supplementary Information:**

The online version contains supplementary material available at 10.3758/s13415-024-01191-y.

Humans and other animals are assumed to have different, parallel decision-making systems at their disposal that solve decision problems in different ways (Kahneman, 2011; Loewenstein & O’Donoghue, 2004; Metcalfe & Mischel, 1999; Milli, Lieder, & Griffiths, 2021; Shiffrin & Schneider, 1977). Some of these systems prioritize speed on behalf of accuracy, yielding quick, but seemingly inaccurate or “irrational” decisions. Other systems prioritize accuracy and yield more “rational” decisions at the cost of lower speed and increased mental resource demand (Dayan, 2014). One particularly simple, yet quick system might be the so-called “Pavlovian” system, responsible for “Pavlovian” or “motivational” biases in behavior (Dayan, Niv, Seymour, & Daw, 2006; Guitart-Masip, Duzel, Dolan, & Dayan, 2014). This system allows the value of cues in the environment—associated with rewards (positive value) or punishments (negative value)—to influence action selection: in the presence of stimuli which signal that a reward can be gained (appetitive cues or “Win cues”), it invigorates behavior and drives more frequent and faster responses. In contrast, in the presence of stimuli which signal that a loss needs to be avoided (aversive cues or “Avoid” cues), it suppresses behavior and leads to fewer and slower responses. Given that these biases seem to be altered in depression (Huys et al., 2016; Nord, Lawson, Huys, Pilling, & Roiser, 2018), traumas (Ousdal et al., 2018), anxiety disorders (Mkrtchian, Aylward, Dayan, Roiser, & Robinson, 2017), and alcohol addiction (Chen et al., 2023; Schad et al., 2020), understanding their role in everyday life might shed light on the etiology and maintenance of these disorders.

The presence of multiple decision systems necessitates an arbitration of which system to rely on in a particular situation, potentially driven by which class of situations (“ecological niche”) each system is most “adaptive” in. Previous frameworks have suggested that different decision systems are selected based on their performance in achieving an optimal tradeoff between speed and accuracy (Daw, Niv, & Dayan, 2005; Keramati, Dezfouli, & Piray, 2011; Milli et al., 2021). Under this framework, Pavlovian biases have been suggested to constitute “default response options” in unfamiliar and/or seemingly uncontrollable environments in which the recruitment of more effortful, “instrumental” control systems does not increase the rate of returned rewards (Dorfman & Gershman, 2019), In such situations, Pavlovian biases might constitute sensible “priors” about which action-outcome contingencies hold in an environment (Moutoussis et al., 2018). Other frameworks have characterized Pavlovian control as an “emergency break” that takes over behavior in presence of particularly large rewards or threats, e.g., when facing a dangerous predator (O’Doherty, Cockburn, & Pauli, 2017). Under such circumstances, the Pavlovian system might trump other systems and induce a global inhibition of all motor effectors, characteristic of the freezing response (Roelofs, 2017; Roelofs & Dayan, 2022; Rösler & Gamer, 2019) and commonly induced by unexpected and surprising events (Schmidt & Berke, 2017; Wessel, 2018; Wessel & Aron, 2017). Notably, freezing seems to occur automatically and outside voluntary control, corroborating its likely “Pavlovian” nature. However, so far, there has been little causal evidence for the claim that such “emergency” high-arousal situations exacerbate Pavlovian biases.

In this study, we initially aimed to test the causal effect of arousal on the size of Pavlovian biases. While many ways of inducing arousal in experimental lab settings exist, most of them can lead to deliberate shifts in response strategy and potentially induce demand characteristics. A promising way circumventing such strategy shifts is the subliminal presentation of arousing stimuli that cannot be consciously perceived by participants. For example, a study that used subliminally presented disgusted faces found these cues to exacerbate biases in a perceptual decision-making task (Allen et al., 2016). As a validation of their subliminal arousal manipulation, they found disgusted faces to affect pupil diameter and heart rate—physiological indices of autonomic arousal. Using emotional faces might be a promising way of modulating Pavlovian biases as suggested by a study that found supraliminally presented angry faces to promote freezing (Ly, Huys, Stins, Roelofs, & Cools, 2014). Motivated by these findings, we used subliminally presented angry and neutral face stimuli to manipulate arousal and to test its effect on the size of Pavlovian biases in behavior. As a manipulation check, we measured pupil diameter, which is commonly interpreted as an index of arousal (Strauch, Wang, Einhäuser, Van der Stigchel, & Naber, 2022) and has in the past been used as a manipulation check in studies using subliminally presented faces (Allen et al., 2016).

To test whether arousal induced by a subliminal presentation of angry faces amplified Pavlovian biases, we combined the orthogonalized Motivational Go/NoGo Task—a task measuring Pavlovian biases in humans—with a subliminal arousal induction while measuring participants’ pupil diameter. We expected Win/Avoid cues featured in the Motivational Go/NoGo Task to induce Pavlovian biases. Furthermore, we hypothesized that subliminally presented angry (compared to neutral) faces would induce heightened arousal, reflected in stronger pupil dilation, which should amplify these biases. We thus expected an interaction between cue valence and the arousal manipulation, with a stronger valence effect, i.e., more Go responses to Win than Avoid cues (reflecting Pavlovian biases), in states of high induced arousal (angry face prime).

Neither responses, reaction times, nor pupil dilation reflected our subliminal arousal manipulation. These null results suggest that the manipulation was ineffective, which meant that we could not assess our preregistered hypotheses. We proceeded with exploratory analyses testing whether features of the task itself were reflected in pupil diameter. A feature that might induce heightened arousal, reflected in pupil diameter, is response conflict between the response required to obtain rewards/avoid punishments and the response triggered by Pavlovian biases (Cavanagh, Eisenberg, Guitart-Masip, Huys, & Frank, 2013; Swart et al., 2018). Participants likely need to recruit additional cognitive and/or physical effort to resolve this conflict (Frank, 2006; Shenhav et al., 2017). We tested whether pupil dilation reflects the *cognitive effort* associated with the suppressing Pavlovian biases, more globally, or the *physical effort* specifically required for invigorating Go responses, which is particularly warranted when overcoming aversive inhibition induced by Avoid cues.

Several recent studies have suggested that pupil diameter reflects cognitive effort associated with the suppression of prepotent responses, e.g., in cognitive conflict tasks, such as the Stroop, Simon, or Flanker task, or in task-switching paradigms (da Silva Castanheira, LoParco, & Otto, 2020; D’Ascenzo, Iani, Guidotti, Laeng, & Rubichi, 2016; Rondeel, Van Steenbergen, Holland, & van Knippenberg, 2015; van der Wel & van Steenbergen, 2018; van Steenbergen & Band, 2013). The very same conflict detection and resolution mechanisms might be required to inhibit Pavlovian biases, i.e., when suppressing Go responses to Win cues or invigorating Go responses in presence of Avoid cues. Previous research has observed increases in midfrontal EEG theta power when participants successfully inhibited Pavlovian biases (Cavanagh et al., 2013; Swart et al., 2018). Other research has found theta power to be correlated with pupil diameter (Dippel, Mückschel, Ziemssen, & Beste, 2017; Lin, Saunders, Hutcherson, & Inzlicht, 2018). In line with these studies, one might expect higher pupil dilations when participants perform a bias-incongruent response, i.e., they successfully inhibit their Pavlovian biases. This pattern would be reflected in an interaction effect between cue valence and response, with stronger dilations for bias-incongruent responses, i.e., Go responses to Avoid cues and NoGo responses to Win cues (Fig. 1E).

**Fig. 1:**
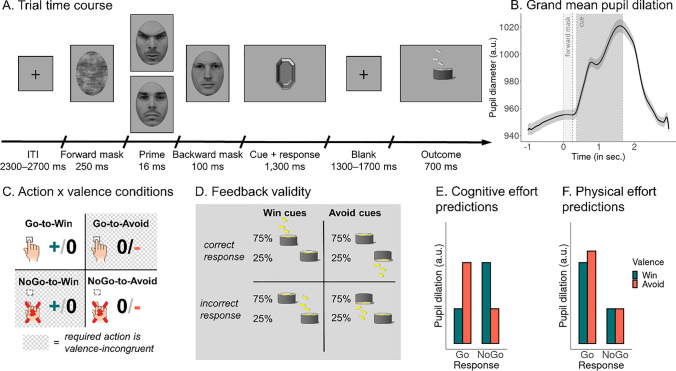
Task design.** A.** *Trial time course*. Each trial starts with a forward mask presented for 250 ms (pixel-permuted version of the neutral prime), a prime stimulus (angry or neutral face) for 16 ms, and a backwards mask (another neutral face) for 100 ms. Participants then see one of four cues and decide whether to respond with a button press (“Go”) or not (“NoGo”). After a variable interval, the outcome (gain, neutral, loss of points) is shown. Face stimuli from the Karolinska Directed Emotional Faces set (Lundqvist, Flykt, & Öhman, 1998). **B.** *Grand mean average of the pupil dilation for all trials of all participants*. Vertical dashed lines indicate the onset of the forward mask (at 0 ms), the prime (at 250 ms), the backwards mask (at 266 ms), the cue onset (at 366 ms), and the cue offset (at 1666 ms). **C.** *Task conditions*. Half of the cues are “Win” cues for which participants can gain points, whereas the other half are “Avoid” cues for which participants can lose points. Orthogonal to the cue valence, one half of the cues requires a Go response (“Go” cues), whereas the other half requires a NoGo response (“NoGo” cues). **D.** *Feedback given cue valence and accura*cy. For half of the cues (“Win cues”), participants receive mostly gains in points (money falling into a can) for correct responses, but no change in point score (a can) for incorrect responses. For the other half (“Avoid” cues), they receive no change in point score (a can) for correct responses, but a loss of points (money falling out of a can) for incorrect responses. **E.** Predictions of per-condition pupil dilation according to the hypothesis that pupil dilation reflects cognitive effort. Dilations are higher for bias-incongruent responses, i.e., Go responses to Avoid cues and NoGo responses to Win cues, irrespective of whether a Go or NoGo response has to be performed. **F.** Prediction of per-condition pupil dilation according to the hypothesis that pupil dilation reflects physical effort. Dilations are higher for Go than NoGo responses and particularly high for Go responses to Avoid cues, for which aversive inhibition must be overcome

Besides cognitive effort, an alternative hypothesis could be that pupil diameter reflects physical effort associated with Go responses. By definition, physical effort is required for performing actions, but not inactions. It is particularly required when performing actions against some obstacle, e.g., lifting a heavy weight, or hindrance, e.g., when held back by a transient state of paralysis as induced by an aversive cue. In contrast, inhibiting action does not require physical effort and does not exhaust humans in the way weight lifting does (Inzlicht, Schmeichel, & Macrae, 2014) and inhibition and physical effort valuation show distinct neural correlates (Klein-Flügge, Kennerley, Friston, & Bestmann, 2016; Wessel & Aron, 2017). Furthermore, physical effort is distinct from cognitive effort in that only actions can be physically effortful, while both actions and inactions can be cognitively effortful if conflicting action tendencies arise that have to be resolved via the recruitment of cognitive control. In the context of the Motivational Go/NoGo Task, which requires only single button presses, “physical effort” might sound like a hyperbole. Still, we chose this term as the decision to act, at all, and the decision to recruit physical effort are likely driven by the same mechanisms (Berke, 2018; Hamid, 2021). Furthermore, animal research has used Go/NoGo tasks akin to our design to study physical effort costs (Hamid et al., 2016; Syed et al., 2016). Finally, also past research in humans has used relatively minor actions, e.g., the speed of single saccades (Manohar et al., 2015) or choice options that require several keyboard presses (Treadway, Buckholtz, Schwartzman, Lambert, & Zald, 2009), as indices of physical effort.

An association between pupil diameter and movement is long established as pupil diameter reliably increases during movement preparation (Richer & Beatty, 1985; Richer, Silverman, & Beatty, 1983; Schacht, Dimigen, & Sommer, 2010). In classic Go/NoGo tasks, dilations are generally higher for Go responses than NoGo responses (Schacht et al., 2010; Van der Molen, Boomsma, Jennings, & Nieuwboer, 1989). We would expect the same pattern in the Motivational Go/NoGo Task. However, going beyond previous research, pupil dilations should be particular high for Go responses to Avoid cues (compared with Go responses to Win cues) because participants have to recruit additional physical effort to overcome aversive inhibition induced by Avoid cues. Thus, there should be an effect of cue valence on pupil dilation selectively for Go responses, which require physical effort, but not for NoGo responses, for which no physical effort is required.

Physical effort and cognitive effort accounts of the pupil response can be dissociated based on NoGo responses to Win cues, which require high cognitive effort (for suppressing Pavlovian biases), but low physical effort (because no movement is prepared). If pupil dilation reflected cognitive effort, dilations should be high for both Go responses to Avoid cues and NoGo responses to Win cues. In contrast, if pupil dilation reflected physical effort, dilations should be high for Go responses to Avoid cues but low for NoGo responses to Win cues, because the latter require no physical effort. Taken together, under a physical effort account of pupil dilation, we would expect a main effect of response on pupil dilation, with stronger dilations during Go than NoGo responses, qualified by an interaction between response and cue valence, with particularly strong dilations for Go responses to Avoid cues (Fig. 1F).

## Methods

### Participants and exclusion criteria

Thirty-five participants (*M*_age_ = 22.37, *SD*_age_ = 2.68, range 18–30; 18 women, 17 men; 27 right-handed, 8 left-handed; 18 with right-eye dominant; 17 with left-eye dominant) took part in this study. The study design, hypotheses, and analysis plan was preregistered on OSF under https://osf.io/ue397. English-speaking participants aged 18–35 years were recruited via the SONA Radboud Research Participation System of Radboud University. Only participants with unimpaired vision or contact lenses were admitted. Exclusion criteria comprised previous neurological treatment, cerebral concussion, brain surgery, or epilepsy. Participants were excluded from all analyses for three (preregistered) reasons: (a) guessing the hypothesis in the debriefing; (b) performance not significantly above chance (tested by using required action to predict performed action with a logistic regression; only participants with *p* < .05 were maintained); and (c) no pupil data on more than 128 trials (50% of trials). None of these criteria applied to any of the participants. Hence, the final sample size for all analyses comprised *N* = 35. This reported research was approved by the local ethics committee of the Faculty of Social Sciences at Radboud University (proposal no. ECSW-2018-171 and ECSW-2019-055) in accordance with the Declaration of Helsinki.

The sample size was not based on a power analysis but on lab availability for this project (4 weeks, April 16 to May 17, 2019), because this study was conducted around several thesis projects. The sample size of *N* = 35 was comparable to previous studies that investigated Pavlovian biases with the same task (Algermissen, Swart, Scheeringa, Cools, & den Ouden, 2022; Swart et al., 2018) and slightly larger than the study that inspired the subliminal arousal priming manipulation (Allen et al., 2016). A post-hoc sensitivity power analysis yielded that, given 35 participants providing 256 trials (thus 8,960 trials in total), and assuming intra-cluster coefficients of 0.04 for responses, 0.14 for RTs, and 0.17 for dilations (all estimated from the data), the effective sample size was *n* = 4,090 for responses, *n* = 1,558 for RTs, and *n* = 1,329 for dilations, respectively, which allows to detect effects of β > 0.04 for responses, β > 0.07 for RTs, and β > 0.08 for dilations (standardized regression coefficients) with 80% power (Aarts, Verhage, Veenvliet, Dolan, & van der Sluis, 2014).

### Procedure

Participants completed a single experimental session that lasted approximately 45 minutes. They provided informed consent, underwent an 9-point eye-tracker calibration, read computerized instructions, and performed four practice trials for each of the four cue conditions. Afterwards, they completed 256 trials of the Motivational Go/NoGo Task. After the task, participants completed measures of trait anxiety (STAI, Form Y-2, 20 items) (Spielberger, Gorssuch, Lushene, Vagg, & Jacobs, 1983) and impulsivity (UPPS-P short version, five subscales, 20 items) (Cyders, Littlefield, Coffey, & Karyadi, 2014), which were part of final year theses written on this data set. At the end, participants went through a funnel debriefing asking them what they thought the hypothesis investigated in the experiment was, whether they used any strategies not contained in the task instructions (and, if yes, describe them), whether they noticed anything special about the task not mentioned in the instructions (and, if yes, describe it), whether they noticed anything special about the face at the beginning of each trial (and, if yes, describe it), whether they recognized the emotions of the face presented very briefly (and, if yes, describe them), and finally, given that there was an angry and a neutral face presented, what they thought the hypothesis investigated in the experiment was. After the completion of the experiment, participants received course credit in compensation plus a performance-dependent candy bar for task accuracy >75%.

### Apparatus

Reporting follows recently suggested guidelines for eye-tracking studies (Fiedler, Schulte-Mecklenbeck, Renkewitz, & Orquin, 2020). The experiment was performed in a dimly lit, sound-attenuated room. The head of the participants was stabilized with a chin rest. The experimental task was coded in PsychoPy 1.90.3 on Python 2.7, presented on a BenQ XL2420Z screen (1920 x 1080 pixels resolution, refresh rate 144 Hz). Participants’ dominant eye was recorded with an EyeLink 1000 tracker (SR Research, Mississauga, Ontario, Canada; sampling rate of 1,000 Hz; spatial resolution of 0.01° of visual angle, monocular recording). The chinrest was placed approximately 90 cm in front of the screen and 70 cm in front of the eye-tracker. Before the task, participants underwent the standard 9-point calibration and validation procedure provided by SR Research, which was repeated until the error for all nine points was below 1°. The screen background during the task was of the same gray (RGB [166, 166, 166]) as during the calibration. Participants were instructed to focus on the fixation cross/center of the screen throughout the task. Manual responses (Go) were performed via the space bar of the keyboard.

### Task

Participants performed 256 trials (split in four blocks of 64 trials each) of an orthogonalized Motivational Go/NoGo learning task (Swart et al., 2018). Unlike classic Go/NoGo tasks in which responses are instructed, in this task, required responses for different cues had to be learned from probabilistic feedback. An equal number of Go and NoGo responses was required. Hence, Go responses were not as prepotent as in classic Go/NoGo tasks. Compared with previous studies using this task, the trial time line of the task was slowed down to reliably measure pupil fluctuations. Each trial started with a series of rapidly presented images used to subliminally induce arousal, followed by a cue indicating the required response and potential outcome of the trial, and finished with the outcome.

The arousal priming manipulation closely followed a procedure previously found effective (Allen et al., 2016). It consisted of a “prime” image presented for 16 ms (two frames), which was either an angry face (image ID AM29ANS; high arousal) or a neutral face (ID AM29NES; low arousal) from the Karolinska Directed Emotional Faces data set (Lundqvist et al., 1998). Hair and background were removed from the face stimulus by cropping it to an elliptical shape (size 281 x 381 pixels; 5.0° x 6.7° visual angle; Fig. 1A). To prevent conscious recognition of the prime stimulus, it was flanked by a forward mask, which was a version of the neutral prime with pixels randomly permuted, presented for 250 ms before the prime, and a backward mask, which was another neutral face taken from the same face data set (ID AM10NES), presented for 100 ms after the prime (Allen et al., 2016). Because of the “subliminal” presentation of the face primes, participants were supposedly unaware of these primes and could not respond to them. Participants were instructed that the presentation of the backward mask served to keep their attention focused on the task.

Next, participants saw one of four cues for 1,300 ms. Unlike the face primes, cues were supraliminally presented gem-shaped stimuli (Fig. 1A) that either required a Go or a NoGo response and either offered the chance to win points (Win cues) or to avoid losing points (Avoid cues). The task was a fully orthogonalized 2 x 2 x 2 design with the factors arousal manipulation via face primes (angry/neutral face), cue valence (Win/Avoid), and required action (Go/NoGo). Participants had to learn from experience whether a cue offered the chance to win points for correct responses (and no change in points for incorrect responses; “Win” cues) or the chance to lose points for incorrect responses (and no change in points for correct responses; “Avoid” cues; Fig. 1C). Also, they needed to learn from trial-and-error whether the cue required a Go response (space bar press) or NoGo response (no press). Cues were of size 300 x 300 pixels (5.3° x 5.3°), presented centrally, set to grayscale, and matched for average luminance and local statistical properties using the SHINE toolbox (Willenbockel et al., 2010). Cue assignment to task conditions was counterbalanced across participants. Each cue was presented 16 times in total (8 times with the high arousal and 8 times with the low arousal prime), with cue presentation interleaved in a pseudo-randomized way (not more than one consecutive cue repetition). Each of the four blocks featured a new set of four cues to prevent ceiling effects in performance and to maximize the time during which participants were (at least partially) unsure about the correct response.

After a variable interstimulus interval (uniform distribution between 1,300–1,700 ms in steps of 100 ms), the outcome was presented for 700 ms. Outcomes consisted in either money falling into a can (positive feedback for Win cues), money falling out of a can (negative feedback for Avoid cues), or simply a can (negative feedback for Win cues/positive feedback for Avoid cues). Feedback validity was 75%, i.e., correct responses were followed by positive feedback and incorrect responses followed by negative feedback on 75% of trials, with the reverse being the case on the remaining 25% of trials (Fig. 1C). Trials finished with a variable intertrial interval (uniform distribution between 2,300–2,700 ms in steps of 100 ms).

### Data preprocessing

#### Behavior

For analyses using RTs, we excluded trials with RTs < 300 ms (in total 36 trials of 8,960 trials; per participant: *M* = 1.01, *SD* = 3.06, range 0–14), because it is implausible that these very fast responses incorporated knowledge about the cue. Note that this step was not preregistered, but the same procedure was used in previous studies in which we used the same task (Algermissen et al., 2022; Swart et al., 2017). Analyses including all RTs led to identical conclusions.

#### Pupil preprocessing

Pupil data were preprocessed in R following previously published pipelines (de Gee et al., 2017; Urai, Braun, & Donner, 2017). First, pupil data was epoched into trials from 1,000 ms before until 2,966 ms after forward mask onset (i.e., until the earliest possible end of the ISI/before possible outcome onset). Note that the preregistration specifies a different time range (1,000 ms before until 1,666 ms after forward mask onset; i.e., exactly until task cue offset) under the assumption of a peak of the pupil response around 1,000 ms (Hoeks & Levelt, 1993). However, in fact, the grand average pupil response in this data peaked at 1,584 ms (Fig. 1B), i.e., close to the end of the pre-registered time window, with per-trial dilations peaking outside the preregistered window on almost half of the trials (assuming symmetric noise on the peak latency). The grand average pupil time course only returned to baseline levels around 3,000 ms after forward mask onset (Fig. 1B). We thus decided to extend the time window until 2,966 ms, i.e., until the earliest possible onset of an outcome (Fig. 1A). After epoching, the timing of blinks and saccades (as automatically detected by the EyeLink software) was extracted. These gaps of missing data were zero-padded by deleting 150 ms (for blinks, 20 ms for saccades) of samples before and after them (as recommended by the EyeLink manufacturer). In addition, we computed the first derivative of the pupil time course and marked abnormally fast pupil changes (absolute values of the z-standardized first derivative higher than 2). If two such marks occurred less than ten samples away from each other, we deleted all samples in-between. Finally, we interpolated missing or deleted samples with linear interpolation and low-pass filtered the data at 6 Hz with a 3-order Butterworth filter. We deleted the first and last 250 ms of each trial to remove edge artifacts caused by the filter. We converted pupil diameter to units of modulation (percent signal change) around the mean of the pupil time series of each block using the grand-mean pupil diameter per block (i.e., 64 trials forming one block). Trials with more than 50% of missing/interpolated data were excluded (in total 166 trials of 8,960 trials; per participant: *M* = 4.74, *SD* = 9.10, range 0–43). Finally, we computed the trial-by-trial pupil baseline as mean pupil diameter in the 500 ms before the onset of the forward mask and the maximal pupil dilation as the maximal value during the 2,966 ms after onset of the forward mask (i.e., until the earliest possible end of the ISI). We then computed the trial-by-trial pupil dilation by subtracting the trial-by-trial pupil baseline from the trial-by-trial maximal dilation.

#### Gaze preprocessing

We analyzed the gaze data similar to the pupil data. After epoching, the timing of blinks and saccades (as automatically detected by the Eyelink software) was extracted. These gaps of missing data were zero-padded by deleting 150 ms (for blinks, 20 ms for saccades) of samples before and after them (as recommended by the Eyelink manufacturer). In addition, we computed the first derivative of the gaze position time course (x- and y-coordinates treated separately) and marked abnormally fast changes in gaze position (absolute values of the z-standardized first derivative higher than 2). If two such marks occurred less than ten samples away from each other, we deleted all samples in-between. We did not apply interpolation for missing gaze data.

### Data analysis

#### Mixed-effects regression models

For regression analyses, we used mixed-effects linear regression (function lmer) and logistic regression (function glmer) as implemented in the package lme4 in R (Bates, Mächler, Bolker, & Walker, 2015). We used generalized linear models with a binomial link function (i.e., logistic regression) for binary dependent variables (Go vs. NoGo responses) and linear models for continuous variables, such as RTs, pupil baseline, and pupil dilation. We used zero-sum coding for categorical independent variables. All continuous dependent and independent variables were standardized such that regression weights can be interpreted as standardized regression coefficients. All regression models contained a fixed intercept. We added all possible random intercepts, slopes, and correlations to achieve a maximal random effects structure (Barr, Levy, Scheepers, & Tily, 2013). *P*-values were computed by using likelihood ratio tests with the package afex (Singmann, Bolker, Westfall, & Aust, 2018). We considered *p*-values smaller than α = 0.05 as statistically significant.

As an initial manipulation check, we fit a mixed-effects logistic regression model with responses (Go/NoGo) as dependent variable and required action (Go/NoGo), cue valence (Win/Avoid), and their interaction as independent variables. Furthermore, we fit an equivalent linear regression model with RTs as dependent variable. For the confirmatory models involving the arousal priming manipulation as independent variable, see Supplementary Material S05. For the confirmatory models involving trial-by-trial pupil dilation as independent variable, see Supplementary Material S06. In further exploratory analyses, we fit a mixed-effects linear regression model with trial-by-trial pupil dilation as dependent variable and response, cue valence, and their interaction as independent variables.

#### Cluster-based permutation tests on pupil data

To test whether the millisecond-by-millisecond pupil time course during a trial differed between conditions, we used cluster-based permutation tests (Maris & Oostenveld, 2007). For cue-locked time courses, we epoched the pre-processed data into trials from −1,000 ms before until 2,966 ms after mask onset, sorted trials into task conditions, and computed the average time course per condition per participant. For response-locked time courses, on trials with Go responses, we epoched the data relative to the trial-specific RT. On trials with NoGo responses, we epoched the data relative to pseudo-RTs computed as the mean RT to Win cues/Avoid cues for a given participant.

We then computed a permutation null distribution by, for 10,000 iterations, randomly exchanging the labels of conditions, computing the mean difference between conditions per participant, computing the overall mean difference between conditions across participants, thresholding this difference at |*t*| > 2, computing the sum of *t*-values for each cluster of adjacent samples above threshold (cluster mass), and retaining the largest cluster mass detected for each iteration. We then compared the empirical cluster mass obtained from the actual data to the permutation null distribution and computed the permutation *p*-value as the number of iterations with a larger cluster mass than the empirical cluster mass divided by the total number of iterations. To correct for pre-trial baseline differences, for each condition, we subtracted the value at time point 0 (also for each iteration in the permutation distribution).

#### Cluster-based permutation tests on gaze data

In line with previous studies reporting freezing of gaze (Rösler & Gamer, 2019), we used the mean gaze position (x- and y-coordinates) in the 500 ms before mask onset (while the fixation cross from the inter-trial interval was on the screen) as a trial-by-trial baseline and compute the absolute deviation (Euclidean distance in pixels) from that baseline for each trial (“gaze dispersion”). This procedure corrects for any drift in the eye-tracking calibration over time. We then computed the mean distance from the pretrial baseline at any timepoint during cue presentation separately for Win and Avoid cues for every participant. We performed a cluster-based permutation test with 10,000 iterations and a cluster-forming threshold of |*t*| > 2 to test for any difference in the distance from the center between Win and Avoid cues.

#### Generalized additive mixed-effects models

Additive models use smooth functions of a set of predictors (i.e., thin plate regression splines) to model a time series. They allow for testing whether the modeled time series differs between conditions. The shape of a smooth function is fitted to the data and can be linear or nonlinear, allowing more flexibility in capturing nonlinear trends over time compared to linear models, which makes them particularly suited for analyzing pupillometry data (Algermissen, Bijleveld, Jostmann, & Holland, 2019; Baayen, Vasishth, Kliegl, & Bates, 2017; van Rij, Hendriks, van Rijn, Baayen, & Wood, 2019). A smooth function regularizes the raw time courses and in this way suppresses high-frequency (trial-by-trial) noise. It also accounts for nonzero auto-correlation between residuals, which is assumed to be zero in linear models.

To test whether the effect of task conditions changed over time, we fit generalized additive mixed-effects models with the trial-by-trial pupil dilation as dependent variable and separate effects of cue repetition (1–16) for each response condition (Go-to-Win, Go-to-Avoid, NoGo-to-Win, NoGo-to-Avoid) as independent variables. We modeled the time course of cue repetition as a factor smooth (which has a similar, but potentially nonlinear effect as adding a random intercept and a random slope) for each participant for each block, allowing for the possibility that condition differences were different in different participants in different blocks (equivalent to a full random-effects structure). We used a scaled *t*-distribution instead of a Gaussian distribution for the response variable in case it led to lower fREML values (which was the case for RTs, pupil baselines, and dilations). In case of significant residual auto-correlation at lag 1 (which was the case for baselines), we added an AR(1) auto-regressive model, with the proportionality constant set to the lag 1-correlation of the residuals from the same model fitted without the AR(1). For all fitted models, we visually checked that residuals were approximately normally distributed using quantile-quantile plots and whether auto-correlation was near zero using auto-correlation plots (van Rij et al., 2019).

### Transparency and openness

We report how we determined our sample size, all data exclusions, all manipulations, and all measures in the study. All data, analysis code, and research materials are available under https://doi.org/10.34973/vh63-k490. The study design, hypotheses, and confirmatory analysis plan were preregistered under https://osf.io/57zjh and updated under https://osf.io/azqjt (extending data collection by 1 week).

We deviated from the preregistration in the definition of the time window in which pupil dilation was defined. This time window was preregistered as spanning 1,000 ms before until 1,666 ms after forward mask onset (i.e., exactly until task cue offset). Given the unexpectedly late peak in the grand average pupil time course, we extended this time window until 2,966 ms after mask onset (i.e., until the earliest possible onset of an outcome). The preregistration also comprised plans for computational models and a deconvolution GLM approach. Because the arousal priming manipulation did not work, these plans were eventually not carried. Finally, we performed exploratory analyses of pupil dilation as a function of task factors (testing physical effort and cognitive effort accounts of pupil diameter), which were not preregistered.

Data were analyzed by using R, version 4.1.0 (R Core Team, 2022). Models were fitted with the package lme4, version 1.1.34 (Bates et al., 2015) and *p*-values computed with the package afex, version 1.3-0 (Singmann et al., 2018). Plots were generated with ggplot, version 3.4.2 (Wickham, 2016).

## Results

### Manipulation checks: Learning and Pavlovian bias

**Fig. 2:**
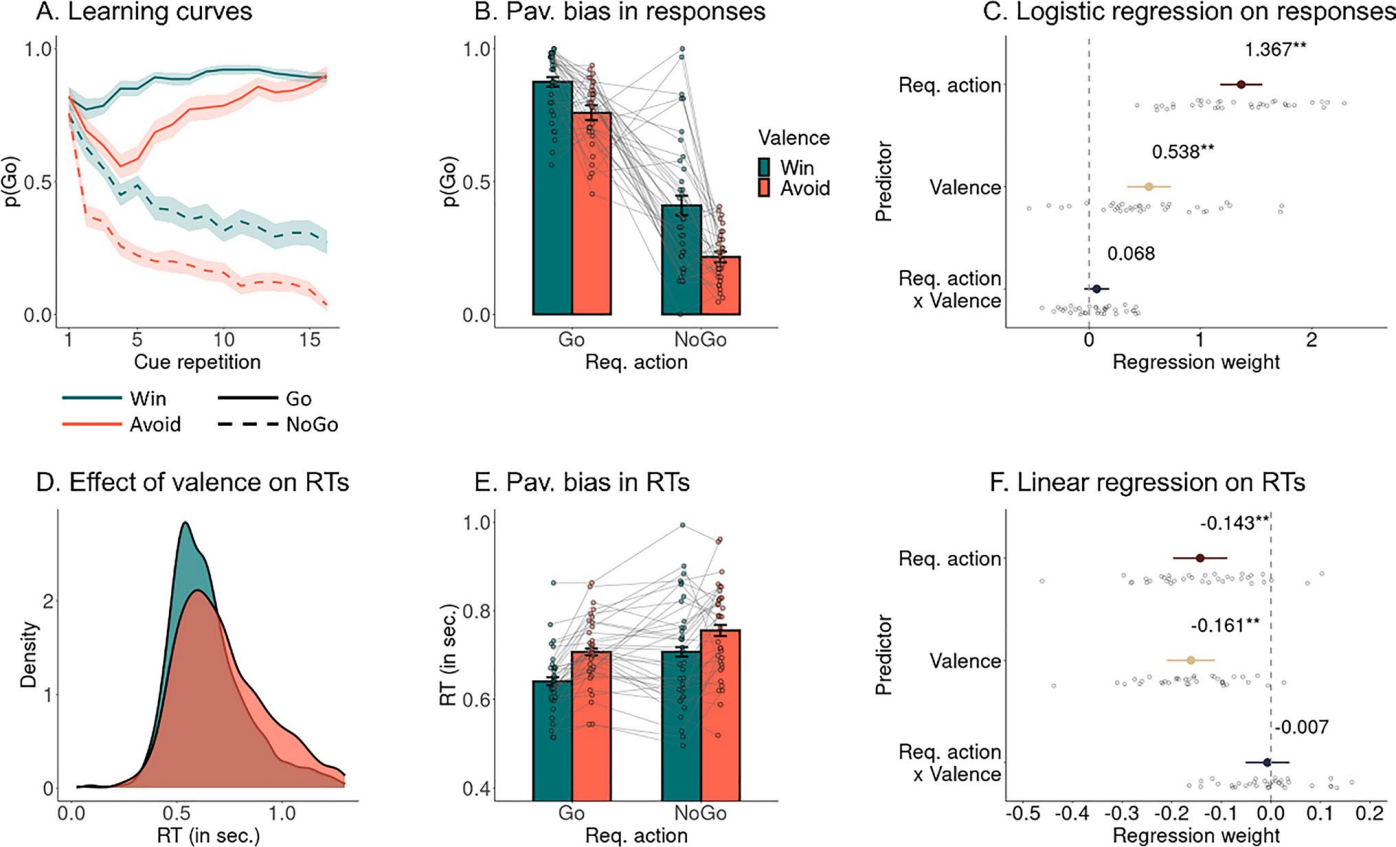
Learning and Pavlovian biases in responses and RTs. **A.** Trial-by-trial proportion of Go responses per cue condition. Participants learn to perform a Go response or not, with significantly more Go responses to Go cues than NoGo cues. Also, they perform significantly more Go responses to Win cues than to Avoid cues, reflecting the Pavlovian bias. Note that participants are initially unaware of the cue valence and have to infer it from (nonneutral) feedback, which explains why the biases only emerge after the first few trials. For the Go-to-Avoid conditions, the biases initially suppress responding, and participants have to subsequently learn to overcome the biases and perform a Go response. This is reflected in the dip in Go responses for Go-to-Avoid cues for trials 1–5 when the negative valence of this cue is learned, and a subsequent rise in Go responding as the correct response to this cue is learned. Error bands are ± SEM across participants. **B.** Proportion of Go responses per cue condition (whiskers are ± SEM across participants, dots indicate individual participants). Participants show significantly more Go responses to Go than NoGo cues (reflecting learning) and significantly more Go responses to Win cues than Avoid cues (indicative of Pavlovian biases). **C.** Group-level (colored dot, 95% CI) and individual-participant (grey dots) regression coefficients from a mixed-effects logistic regression of responses on required action, cue valence, and their interaction. **D.** Distribution of raw RTs separately per cue valence. **E.** Mean RTs per cue condition. Participants show significantly faster (correct) Go responses to Go than (incorrect) Go responses to NoGo cues and significantly faster responses to Win cues than Avoid cues (indicative of Pavlovian biases). **F.** Group-level and individual-participant regression coefficients from a mixed-effects linear regression of RTs on required action, cue valence, and their interaction

First, in line with the preregistration, we performed manipulation checks to replicate effects typically found with this task (Algermissen et al., 2022; Swart et al., 2018). We fit a mixed-effects logistic regression with responses (Go/NoGo) as dependent variable and required action (Go/NoGo) and cue valence (Win/Avoid) as well as their interaction as independent variables (see Supplementary Material S01 for an overview of all regression results; see Supplementary Material S02 for means and standard deviations per condition). Participants performed significantly more (correct) Go responses to Go cues than (incorrect) Go responses to NoGo cues (required action), *b* = 1.367, 95% CI [1.178, 1.556], χ^2^(1) = 66.523, *p* < .001, indicating that participants successfully learned the task (Fig. 2A-C). Overall, participants showed 82% accuracy for Go cues (*SD* = 10%, range 63–95%) and 69% accuracy for NoGo cues (*SD* = 15%, range 37–96%; see also Supplementary Material S02 for per condition mean response rates). Also, participants performed more Go responses to Win than to Avoid cues (cue valence), *b* = 0.538, 95% CI [0.341, 0.734], χ^2^(1) = 20.986, *p* < .001, reflecting Pavlovian biases (Fig. 2A-C). The interaction between required action and valence was not significant, *b* = 0.068, 95% CI [−0.044, 0.181], χ^2^(1) = 1.348, *p* = .246, providing no evidence that Pavlovian biases were stronger for Go or for NoGo cues.

Furthermore, we fit a mixed-effects linear regression with RTs as dependent variable and again required action, cue valence, and their interaction as independent variables. This analysis was omitted in the preregistration, but in line with previous studies (Algermissen et al., 2022). RTs were only available for Go responses. Participants were faster at correct responses (to Go cues) than incorrect responses (to NoGo cues; required action), *b* = −0.143, 95% CI [−0.197, −0.088], χ^2^(1) = 20.446, *p* < .001 (Fig. 2D-F). Also, they were faster at performing responses to Win than to Avoid cues (cue valence), *b* = −0.143, 95% CI [−0.197, −0.088], χ^2^(1) = 27.329, *p* < .001, again reflecting the Pavlovian biases (Fig. 2D-F). For the time courses of both effects over trials within a block, see Supplementary Material S03. The interaction between required action and valence was not significant, *b* = −0.007, 95% CI [−0.051, 0.037], χ^2^(1) = 0.083, *p* = .773, providing no evidence that Pavlovian biases were stronger for correct responses (to Go cues) or incorrect responses (to NoGo cues). Pavlovian biases in responses and RTs were not correlated with participants’ trait anxiety or impulsivity scores, providing no evidence for stronger or weaker biases in more impulsive/more anxious individuals (see Supplementary Material S04). Taken together, these results corroborate that participants learned the task and exhibited Pavlovian biases.

### Exploratory analyses: Freezing of gaze induced by Avoid cues

Previous research on humans and animals has investigated the phenomenon of “freezing,” i.e., temporarily reduced body motion in presence of a threat (Blanchard, 2017; Roelofs, 2017). Freezing in humans is typically measured via reductions in heart rate (Hashemi et al., 2019; Klaassen et al., 2021) and bodily mobility (Ly et al., 2014) tracked with a stabilometric force-platform that records spontaneous fluctuations in body sway. Recently, it has been suggested that freezing might also affect gaze, with a stronger center bias and less visual exploration while participants prepare a response to avoid an electric shock (Merscher & Gamer, 2024; Merscher, Tovote, Pauli, & Gamer, 2022; Rösler & Gamer, 2019). We tested whether a similar freezing of gaze pattern occurred during the presentation of Avoid compared to Win cues in the context of the Motivational Go/NoGo Task, testing for a difference in the absolute distance from the center of the screen (“gaze dispersion”) between trials with Win and Avoid cues.

**Fig. 3:**
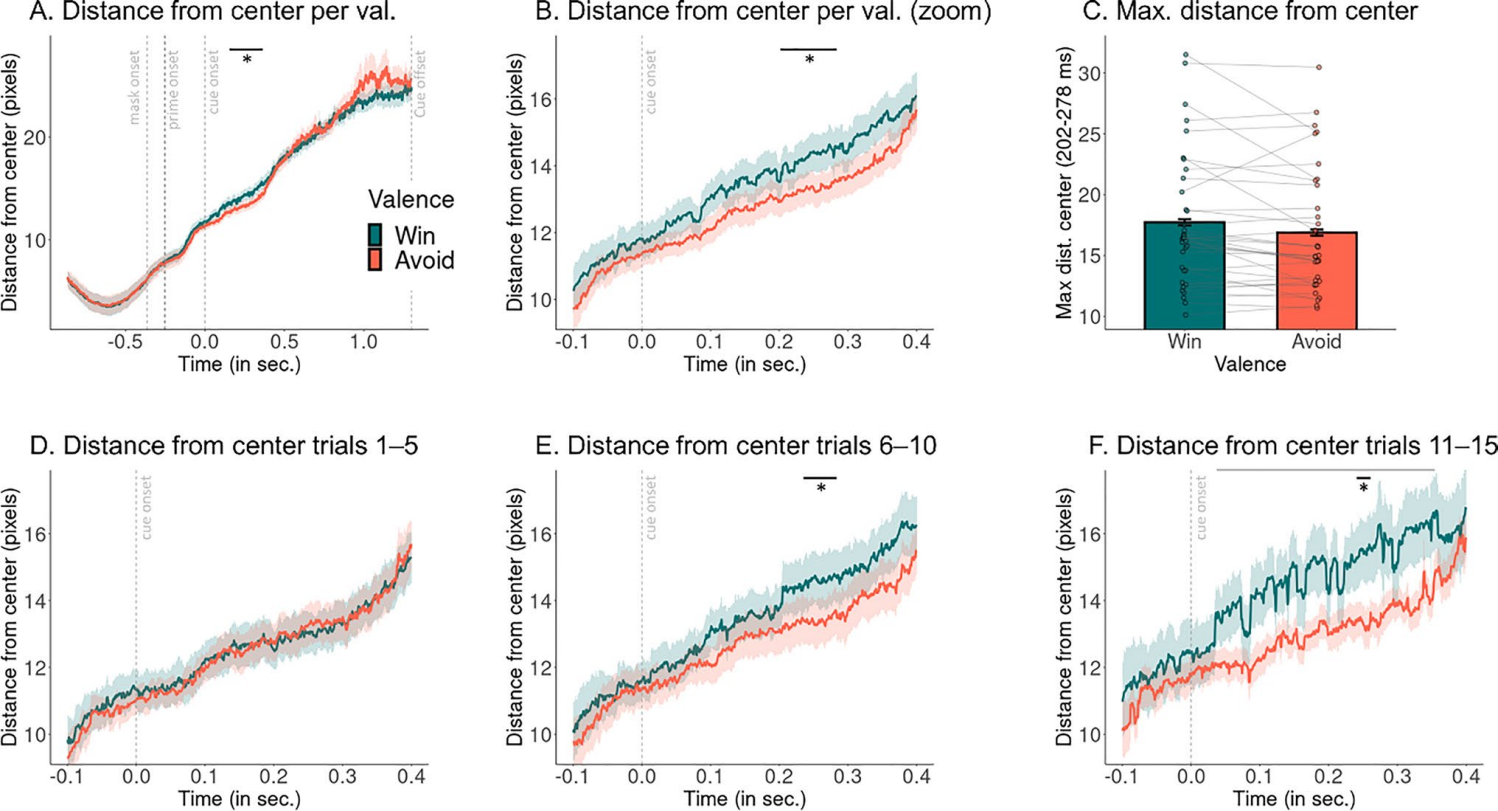
Freezing of gaze induced by Avoid cues.** A.** Mean distance from the gaze position during the trial-by-trial baseline (“center”). Vertical dashed lines indicate the onset of the forward mask (at −366 ms), the prime (at −266 ms), the backwards mask (at −250 ms), the cue onset (at 0 ms), and the cue offset (at 1300 ms). Distance increases with time. Around 202–278 ms after cue onset, distance from the center is lower on trials with Avoid cues compared with trials with Win cues. **B.** Same as panel A but zoomed into the time range of −100 to 400 ms after cue onset. **C.** Maximum distance from the pretrial baseline (whiskers are ± SEM across participants, dots indicate individual participants) averaged for Win and Avoid cues for each participant. Distance is lower on trials with Avoid cues compared with trials with Win cues. **D-F.** Same as panel B but computed for subsets of trials. While freezing of gaze is absent on the first five cue repetitions when participants are not yet fully aware of the cue valence (see learning curves in Fig. 2A), with no cluster above threshold (**D**), the freezing of gaze bias emerges on cue repetitions 6–10 (**E**; *p* = .009; cluster above threshold 242–281 ms after cue onset) and becomes even stronger on cue repetitions 11–15 (**F**; multiple disconnected clusters above threshold between 55–353 ms after cue onset, grey horizontal line; largest cluster above threshold from 245–261 ms, *p* = .023, black horizontal line)

A cluster-based permutation test in the time range of 0–500 ms after cue onset was significant (*p* = .024, two-sided; driven by a cluster above threshold 202–278 ms after cue onset; Fig. 3A, B). Distance from the center was lower on trials with Avoid cues than on trials with Win cues, in line with the idea of “freezing of gaze” induced by Avoid cues. Computing the maximal distance from the center in this time window for every trial, averaging distances for Win and Avoid cues per participant, and then averaging across participants confirmed this difference (Fig. 3C). Importantly, there was no difference in gaze dispersion between Win and Avoid cues on the first five repetitions of a cue, i.e., when participants were not fully aware of cue valence yet (Fig. 3D; no cluster above threshold), but this difference only emerged on cue repetitions 6–10 (Fig. 3E; *p* = .009, cluster above threshold from 242–281 ms after cue onset) and became stronger for cue repetitions 11–15 (Fig. 3F; multiple disconnected clusters above threshold between 55–353 ms after cue onset; largest cluster above threshold from 245–261 ms, *p* = .023).

In summary, we found evidence for freezing of gaze induced by Avoid cues, with lower gaze dispersion on trials with Avoid cues compared to trials with Win cues. This difference in gaze dispersion only emerged after learning the aversive nature of the cues.

### Confirmatory analyses: No effect of the arousal manipulation on responses, RTs, or pupil dilation

In preregistered confirmatory analyses, we tested whether the arousal manipulation (angry/neutral face primes) affected the proportion of Go/NoGo responses, RTs, or pupil dilation, either as a main effect or in interaction with cue valence, required action, or the trial-by-trial pupil dilation. In brief, the arousal manipulation had no effect on any dependent measure, also not in interaction with other task factors. These findings suggest that the manipulation was ineffective and participants did not process the face primes, neither consciously nor subconsciously. We report the full results of all pre-registered confirmatory as well as additional exploratory analyses in Supplementary Material S05, in which we also discuss reasons for why the manipulation might have been ineffective.

### Exploratory analyses: Stronger trial-by-trial pupil dilations for Go responses, especially to Avoid cues

We measured trial-by-trial fluctuations in pupil dilations for two reasons. First, pupil dilation is commonly interpreted as an index of arousal, which could reveal whether the arousal manipulation via angry/neutral face primes was effective. Second, pupil dilation can index other arousal-related processes such as cognitive and physical effort, which might be required on incongruent trials in order to suppress Pavlovian biases. Specifically, cognitive effort is required for conflict detection and resolution on all incongruent trials, i.e., both when inhibiting Go responses to Win cues and when invigorating Go responses to Avoid cues. Higher pupil dilations on incongruent trials should be reflected in an interaction effect between cue valence and required action, with stronger dilations for NoGo than Go responses to Win cues, but stronger dilations for Go than NoGo responses to Avoid cues (Fig. 1E). In contrast, physical effort is only required for making Go responses, and particular effort might be required for Go responses to Avoid cues when aversive inhibition induced by Avoid cues has to be overcome. Hence, if pupil dilation specifically reflected physical effort, one would expect a main effect of response, with stronger dilations for Go than NoGo responses, qualified by an interaction effect between response and cue valence, with stronger dilations for Go responses to Avoid than to Win cues, but no difference between Win and Avoid cues for NoGo responses (Fig. 1F). To test these two accounts, we performed exploratory, non-preregistered analyses of trial-by-trial fluctuations in pupil dilation, operationalized as the maximum dilation between forward mask onset and minimal possible ITI offset (i.e., 2,966 ms after the forward mask) minus the premask baseline (−500 to 0 ms before the mask), as a function of the executed response (Go/NoGo) and cue valence (Win/Avoid).

**Fig. 4:**
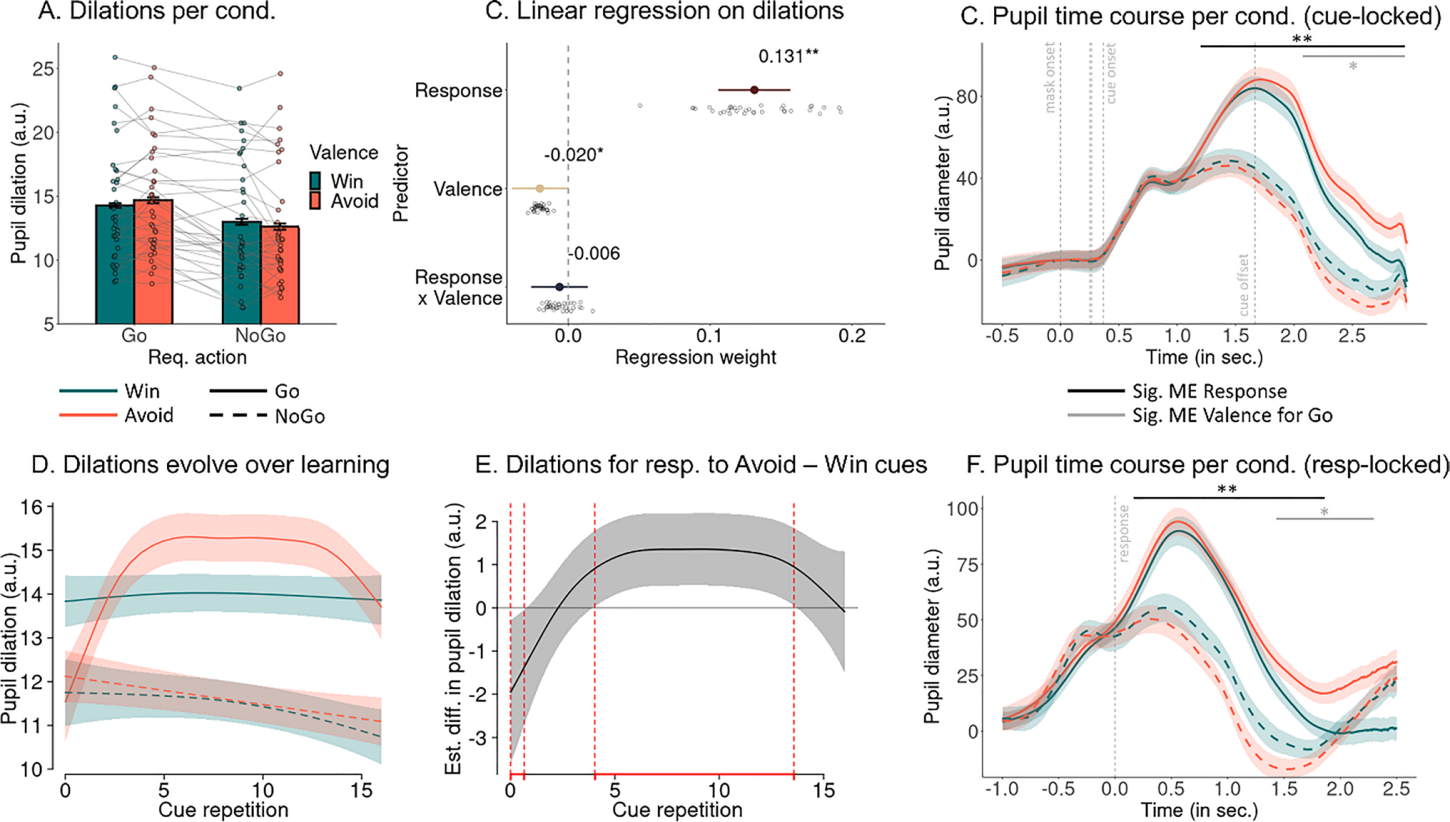
Pupil dilations as a function of the response and cue valence.** A.** Mean pupil dilation per response and cue valence. Dilations are significantly higher for Go than NoGo responses and significantly higher for Go responses to Avoid cues than responses to Win cues. **B.** Group-level (colored dot, 95% CI) and individual-participant (grey dots) regression coefficients from a mixed-effects linear regression of dilations on response, cue valence, and their interaction. There are significant main effects of response and cue valence, but the interaction is not significant. **C.** Pupil time course within a trial locked to forward mask onset per response per cue valence (mean ± SEM across participants; baseline-corrected). Vertical dashed lines indicate the onset of the forward mask (at 0 ms), the prime (at 250 ms), the backwards mask (at 266 ms), the cue onset (at 366 ms), and the cue offset (at 1666 ms). The pupil dilates significantly more on trials with Go responses than on trials with NoGo responses starting 1,190 ms after forward mask onset (black horizontal line). Furthermore, the pupil dilates significantly more sustainedly for responses to Avoid than to Win cues, starting 2,157 ms after forward mask onset (grey horizontal line). See Supplementary Material S08 for a version without baseline correction. **D.** Time course of dilations over cue repetitions (mean ± SE) as predicted from a generalized additive mixed-effects model (GAMM), separated by response and cue valence. Dilations are significantly stronger on trials with Go responses than on trials with NoGo responses through blocks. Furthermore, dilations are significantly stronger for responses to Avoid cues than to Win cues from cue repetition 3 to 13, putatively reflecting heightened effort recruitment on trials with Avoid cues to overcome aversive inhibition. **E.** Difference line between dilations on trials with responses to Avoid cues minus Win cues. Areas highlighted in red indicate time windows with significant differences. **F.** Pupil time course within a trial locked to RTs (for NoGo responses: locked to mean RT for responses to Win/Avoid cues per participant) split by response and cue valence (mean ± SEM across participants; baseline-corrected). The vertical dashed lins indicates the RT (at 0 ms). Note that neither cues nor outcomes are systematically time-locked to RTs and thus not represented in this figure. The pupil dilates significantly more on trials with Go responses than on trials with NoGo responses starting 144 ms after the RT (black horizontal line). Furthermore, the pupil dilates significantly more sustainedly for responses to Avoid than to Win cues, starting 1,516 ms after the RT (grey horizontal line). This latter difference continues up until the outcome-induced pupil dilation (right end of figure)

We observed a significant main effect of Go/NoGo responses on trial-by-trial pupil dilation, *b* = 0.112, 95% CI [0.084, 0.140], χ^2^(1) = 38.769, *p* < .001, with much stronger pupil dilations on trials with Go responses compared to trials with NoGo responses (Fig. 4A, B). Furthermore, there was a significant main effect of valence, *b* = −0.020, 95% CI [−0.040, −0.001], χ^2^(1) = 4.007, *p* = .045, with stronger dilation for Avoid than Win cues (Fig. 4A, B). The interaction between performed action and valence was not significant, *b* −0.006, 95% CI [−0.026, 0.014], χ^2^(1) = 0.356, *p* = .551. However, the pattern displayed in Fig. 4A was suggestive of an interaction effect, with higher dilations for Avoid than Win cues only for Go responses, with this pattern reversing for NoGo responses. This observation was confirmed when using post-hoc *z*-tests, which yielded a significant effect of valence only for Go responses, *z* = 1.974, *p* = .048, but not for NoGo responses, *z* = 0.915, *p* = .360. Complementary analyses with dilations as independent variable and responses as dependent variable reproduced these results (see Supplementary Material S06).

These results are in line with a physical effort account of pupil dilation, with stronger dilations for Go responses and particularly for responses to Avoid cues, which require overcoming aversive inhibition. We followed up on this inconsistency between regression results (Fig. 4B) and the pattern observed when plotting the data (Fig. 4A) with more fine-grained analyses of the pupil time courses during trials.

### Exploratory analyses: More sustained pupil dilations for Go responses to Avoid cues

The previous analyses focused on the trial-by-trial peak of the pupil time course, which is a frequently used summary statistic of the pupil time course. However, it does not capture any variation beyond the peak height, such as condition differences in peak timing or in how sustained the peaks are. Given the above-reported inconsistency between regression results and patterns observed when plotting the data, as a more sensitive measure of condition differences, we tested for such differences in the millisecond-by-millisecond pupil time course using cluster-based permutation tests (Strauch et al., 2022). We corrected for any pre-onset baseline differences (for results without baseline correction, see Supplementary Material S08).

The pupil was significantly wider on trials with Go compared to trials with NoGo responses, *p* < .001, driven by a cluster above threshold from 1,190–2,966 ms after mask onset (i.e., until the end of the testing window; Fig. 4C). Within this time window, the pupil was significantly wider for Go responses to Avoid cues than Go responses to Win cues, *p* = .035, driven by a cluster above threshold from 2,157–2,966 ms (i.e., until the end of the testing window; Fig. 4C). There was no significant difference between NoGo responses to Win and to Avoid cues, *p* = 1, with no cluster above threshold.

The difference between Go responses to Avoid and Win cues occurred rather late (2,157–2,966 ms), i.e., after the peak of the grand mean pupil response (at 1,591 ms) and after the task cue had already disappeared (i.e., after 1,666 ms). Responses to Avoid cues were most prominently associated with more sustained, rather than stronger pupil dilations. Despite the late timepoint of this condition difference, due to the sluggishness of the pupil response, it might reflect differences in cognitive processing occurring much earlier, i.e., during cue processing and response selection. Note that this difference occurred much later than differences in gaze dispersion between Avoid and Win cues (i.e., 202–278 ms after cue onset); freezing of gaze and differences in pupil dilation are thus unlikely to confound each other. The difference between responses to Avoid and Win cues also occurred when locking the pupil time course to the response (RT) instead of the mask onset, with stronger dilations during Go than NoGo responses (144–1,934 ms relative to response, *p* < .001) and, within this window, stronger dilations during Go responses to Avoid cues compared with Win cues (1,516–1,934 ms, *p* = .015; Fig. 4F). The latter difference in fact continued up until the outcome-induced dilation as visible in outcome-locked analyses (Supplementary Material S09). For results without baseline-correction, see Supplementary Material S08. For associations between task factors and outcome-locked pupil dilations, see Supplementary Material S09.

These results confirmed the pattern of Fig. 4A, with a strong main effect of the executed response on pupil dilation, qualified by particularly strong and sustained pupil dilations for Go responses to Avoid cues. They are in line with a physical effort account of pupil dilation (Fig. 1F), with particularly high physical effort recruitment when overcoming aversive inhibition induced by Avoid cues. However, they are not in line with a cognitive effort account of pupil dilation, because NoGo responses to Win cues, which require response inhibition and thus cognitive effort, were not associated with strong pupil dilations. Notably, effort requirements should vary over time. They should start once participants have recognized the cue valence and the response required for a given cue, subsequently rise while participants try to counteract their Pavlovian biases, but eventually diminish again once the task is well learned. Hence, next, we tested for condition differences in the dilation time course within task blocks and how these changed with learning.

### Exploratory analyses: Stronger dilations for Go responses to Avoid cues arise and vanish again with learning

Pavlovian biases and their effective suppression depends on participants learning the cue valence and required response, recognizing the demand for physical effort recruitment to suppress aversive inhibition. Participants are initially unaware of the correct response or cue valence and thus do not recruit particular physical effort to invigorate Go responses to Avoid cues (see learning curve per cue in Fig. 2A). As they become more certain about which response to perform, physical effort recruitment might increase, particularly for the cues they have learned to be Avoid cues. With further learning, response selection becomes more certain and the instrumental system dominates the Pavlovian system, requiring less effort with increasing practice. As a result of these two antagonistic trends, an inverted U-shape, with maximal effort recruitment at intermediate stages of learning, could be expected. To test this hypothesis, we fit generalized additive mixed-effects models to participants’ trial-by-trial pupil dilations, testing whether the time course of pupil dilations (modeled via the cue repetition, 1–16) differed between conditions.

The model suggested significantly higher pupil dilations for Go than NoGo responses throughout learning (repetitions 1–16), parametric term *t*(5.54, 7.45) = 14.585, *p* < .001, smooth term *F*(1.32, 1.56) = 2.340, *p* = .165. Furthermore, pupil dilations were significantly stronger for Go responses to Avoid cues than to Win cues between cue repetitions 3 to 13 (and lower around cue repetition 1), parametric term *t*(5.75, 7.67) = 3.039, *p* = .002, smooth term *F*(3.39, 4.16) = 3.483, *p* = .007 (Figs. 4D, E). Note how this time course is mirroring the learning curve for Go-to-Aoid cues (Fig. 2A). See Supplementary Material S07 for results showing that this pattern held independently of other factors affecting pupil dilations for Go responses, such as accuracy, response speed, and response repetition.

These results indicate that stronger dilations for Go responses to Avoid compared with Win cues occurred specifically at intermediate stages of learning, when overcoming aversive inhibition has become driven by past experiences, but not sufficiently practiced yet.

## Discussion

In this study, we tested whether subliminally induced arousal modulated Pavlovian biases in an orthogonalized Motivational Go/NoGo task and whether measured fluctuations in arousal indexed via pupil dilation reflected processes involved in inhibiting those biases. Win versus Avoid cues induced strong Pavlovian biases in both responses and RTs, with faster and more Go responses to Win compared to Avoid cues. The aversive nature of the Avoid cues even elicited a brief “freezing” of gaze, with less gaze dispersion from the center for Avoid compared to Win cues. However, neither responses, nor RTs, nor pupil dilations showed any effect of the arousal priming manipulation, suggesting that this manipulation—although successfully used in past research (Allen et al., 2016)—was ineffective (for a discussion of these findings, see Supplementary Material S05).

Exploratory analyses showed that measured fluctuations in trial-by-trial pupil dilation reflected participants’ responses, specifically the physical effort they recruited to exert a Go response: Stronger dilations occurred on trials with Go responses, with particularly strong and sustained dilations for responses to Avoid cues that were performed against the hindrance induced by Pavlovian biases. There were no comparable pupil responses for trials in which participants inhibited responses to Win cues, which also required the suppression of Pavlovian biases. Thus, pupil dilations do not reflect response conflict or cognitive effort associated with resolving such conflict on “incompatible” trials, but selectively the physical effort required for overcoming the aversive inhibition induced by Avoid cues. Notably, stronger pupil dilations for Go responses to Avoid cues only emerged with learning, indicative that they do not reflect motor processes per se, but the specific physical effort demands required. Taken together, these results are in line with an account of pupil dilation reflecting physical (but not cognitive) effort investment. Beyond previous literature on conflict detection and response suppression in the context of Pavlovian biases (Cavanagh et al., 2013; Swart et al., 2018), these results highlight another cognitive capacity required to manage Pavlovian biases, namely response invigoration against adversities.

### Freezing of gaze by Avoid cues

Avoid cues robustly reduced response rates and slowed reaction times. Note that strong aversive Pavlovian biases are usually absent in variants of the Motivational Go/NoGo Task that separate Pavlovian cues and the response window in time (Guitart-Masip et al., 2012; Queirazza, Steele, Krishnadas, Cavanagh, & Philiastides, 2023). Hence, the instruction to respond immediately to the appearance to the cue seems necessary for observing these biases in behavior. Only in such a variant does it become possible to study the mechanisms by which participants overcome an aversive bias.

Beyond Pavlovian biases in responses and RTs, we also found cue valence to affect gaze position: During the cue presentation, participants’ gaze showed less dispersion from the center of the screen for Avoid cues compared with Win cues in a time range around 200–280 ms after cue onset, with differences becoming stronger with learning. It is notable that, compared with previous studies reporting freezing of gaze (Merscher & Gamer, 2024; Merscher et al., 2022; Rösler & Gamer, 2019), the reduction in gaze dispersion observed in this study was temporally and spatially very constrained. This difference likely arises from differences in the experimental set up. Previous studies encouraged participants to visually explore photos of natural scenes while they prepared for a button press to prevent an electric shock. In contrast, in our task, participants were instructed to maintain fixation at the center of the screen while an aversive cue signaling the chance of losing points was presented. This might explain why the freezing of gaze effect in our study was much smaller than in previous studies, reflecting differences in only a few pixels instead of hundreds of pixels, with a duration of less than 100 ms.

We also considered the possibility that reduced gaze dispersion for Avoid cues did not reflect automatic “Pavlovian” effects but rather deliberate, strategic response adjustments for such cues. Notably, the freezing effect on gaze occurred within less than 300 ms after cue onset, which is faster than typical EEG correlates of task engagement (e.g., the P300 event-related potential). Differences in pupil dilation, which we interpret as reflecting differences in physical effort exertion, occurred more than 2,000 ms later, suggesting that effort-related processes were separate from this early freezing of gaze. Taken together, freezing of gaze likely reflects early, automatic Pavlovian processes, which might be followed by later deliberate changes in task engagement to counteract the freezing, rather than vice versa. Beyond high-level differences in task engagement, differences between cue valence conditions are unlikely to be driven by low-level visual properties, because we matched all cues for average luminance and counterbalanced the assignment of cues to task conditions across participants. Lastly, we observed that this freezing of gaze phenomenon was not yet present on the first five occurrences of a cue when cue valence had not been learned but emerged only in the middle of blocks when participants had become aware of the cue valence. These considerations suggest that the observed freezing of gaze is specifically related to the learned valence of the cues and reflects early automatic processes rather than later strategic processes that aim to overcome aversive inhibition.

Our results corroborate recent evidence that freezing does not merely affect limb movements but also the oculomotor system. Past research has shown that the chance to gain rewards speeds up saccades (Manohar et al., 2015; Shadmehr, Reppert, Summerside, Yoon, & Ahmed, 2019; Tachibana & Hikosaka, 2012), a process sensitive to dopamine and likely implemented by the direct pathway of the basal ganglia (Grogan, Sandhu, Hu, & Manohar, 2020; Kawagoe, Takikawa, & Hikosaka, 1998). Conversely, the indirect pathway in the basal ganglia seems responsible for the suppression of eye movements in presence of low-value objects (Amita & Hikosaka, 2019; Kim, Amita, & Hikosaka, 2017), a role it might also play for negative events, such as aversive cues and threats of punishment. Overall, these findings suggest a more principled role of the basal ganglia in modulating the vigor of eye movements as a function of incentives (Park, Coddington, & Dudman, 2020; Turner & Desmurget, 2010). Our results contribute to this literature by showing how the oculomotor system can give insights in reward and punishment processing not only in animals but also in humans (Shadmehr et al., 2019).

### Pupil dilation reflects physical effort expenditure in a graded fashion

Apart from gaze, pupil dilations also reflected aspects of the Motivational Go/NoGo Task. The biggest effect on pupil dilations was caused by responses, with much stronger pupil dilations for Go than for NoGo responses. This finding concords with a large body of literature reporting stronger pupil dilations under movement preparation, movement execution, and effort exertion (Beatty, 1982; Bijleveld, Custers, & Aarts, 2009; da Silva Castanheira et al., 2020; Kurniawan, Grueschow, & Ruff, 2021; van der Wel & van Steenbergen, 2018; Zénon, Sidibé, & Olivier, 2014). However, it is still an open question which specific processes drive these previously observed response-related pupil dilations. Some studies have argued that such response-related pupil dilations constitute an epiphenomenon of motor movements, i.e., a signal that qualitatively reflects whether a movement is executed or not in an all-or-nothing fashion (Richer & Beatty, 1985; Richer et al., 1983). Alternatively, pupil dilations have been suggested to reflect the effort that is required to execute a response in a more graded, continuous fashion (da Silva Castanheira et al., 2020; van der Wel & van Steenbergen, 2018). Our results concur with the latter interpretation given that we found particularly strong and sustained dilations for responses to Avoid cues, which is plausible, because Avoid cues induce aversive inhibition, which might require particular effort to overcome. This effect was not constant as expectable for a motor artifact but changed systematically with learning. Further supplementary analyses revealed particularly strong dilations for slow responses, which might reflect conflict and effort recruitment, as well. We discuss these findings in the following. Lastly, we found no comparable increase in pupil dilations for NoGo responses to Win cues, arguing against a cognitive effort account of pupil dilation.

Higher pupil dilations during responses to Avoid than to Win cues specifically reflect effort demands, which dynamically change as a function of learning. Differences between Avoid and Win cues occurred specifically in the middle of each block, i.e., after participants were made aware of the cue valence, but before they had fully learned the correct response. At the beginning of each block, new cues were introduced, and until participants had experienced a win or loss in points, they could not know the cue valence. Thus, until the aversive nature of Avoid cues had been experienced, these cues did not induce aversive inhibition nor did they motivate additional effort recruitment. Similarly, little effort was required at the end of blocks when the instrumental learning system had acquired reliable action values that were unlikely to be “swayed” by Pavlovian biases (Dorfman & Gershman, 2019). Additionally, at the end of each block, the experienced rate of punishments had become lower due to increased accuracy, which in turn might have lowered the aversive value of the cues and reduced aversive inhibition. In summary, effort was recruited only after the aversive nature of cues had become clear and only until responses to them became well-learned, concurring with the interpretation of pupil dilation as reflecting effort recruited to overcome aversive inhibition.

Another piece of evidence suggesting that pupil dilations reflect effort recruitment in a continuous fashion is the finding that dilations were stronger for slower compared to faster responses (see Supplementary Materials S06 and S07). Slow responses are often interpreted as reflecting action selection against difficulties, involving effortful cognitive control to resolve conflict (Frank, 2006). The link between dilations and responses was particularly strong for incorrect Go responses (to NoGo cues), which were slower than correct responses (to Go cues), implying that these do not reflect “impulsive” errors, but rather deliberate choices made in spite of previous feedback providing evidence against Go responses. Such slow, incorrect responses might have required particularly high levels of physical effort to trigger a Go response against competing instrumental processes suggesting a NoGo response. Notably, this type of effort was not associated with relatively faster, but slower responses, reflecting situations where eventual Go responses result sequentially from conflict detection and subsequent effort recruitment. Hence, in the context of this task, the recruitment of “physical effort” or “vigor” was not in the service of speeding up responses but instead of executing responses in the first place.

One might wonder how our findings relate to the literature that links pupil dilation to cognitive effort associated with response conflict or task switching (van der Wel & van Steenbergen, 2018). Studies of these phenomena have usually employed paradigms that feature choices between several “Go” responses. Boosting a slow, more controlled response over an automatic, prepotent response might require inhibiting the latter, but also could be implemented by invigorating of the former, which likely involves some form of physical effort. In contrast, tasks requiring pure response inhibition, e.g., classic Go/NoGo tasks, are experienced as cognitively effortful (Dixon & Christoff, 2012) but arguably require little physical effort. In such tasks, pupil dilations are smaller for effortful, controlled NoGo responses compared to prepotent Go responses, which suggests that response conflict and cognitive effort associated with the inhibition of prepotent responses are not sufficient to drive pupil dilations (Schacht et al., 2010; Van der Molen et al., 1989). Together with our results, these findings suggest that pupil dilation is more tightly linked to the invigoration of slow, controlled, deliberate responses, rather than response conflict or cognitive effort per se. This link is only visible in specific paradigms, such as the Motivational Go/NoGo Task, that create response conflict but dissociate physical effort from cognitive effort requirements. Note however that some paradigms requiring cognitive effort, but no particular physical effort, such as mental arithmetic or problem solving tasks, have yielded increased pupil dilations (Hess & Polt, 1964; Kahneman, 1973). Future research will have to identify which specific task features induced these dilations. What we conclude from this study is that response conflict and inhibition alone, such as NoGo responses to Win cues in this task, are not sufficient to drive strong pupil dilations.

### Putative neural mechanisms of aversive biases and their suppression

The link between pupil dilation and physical effort is corroborated by direct recordings from neurons in the locus coeruleus, the major source of noradrenaline in the brain, which strongly correlates with pupil dilations (Joshi, Li, Kalwani, & Gold, 2016; Strauch et al., 2022). Such direct recordings in monkeys have linked noradrenaline levels to physical effort expenditure (Bornert & Bouret, 2021; Varazzani, San-Galli, Gilardeau, & Bouret, 2015). Specifically, one study recorded activity from the substantia nigra and locus coeruleus, the primary sources of dopamine and noradrenaline, while monkeys performed a reward/effort trade-off task involving a grip forcer (Varazzani et al., 2015). Dopamine reflected expected value and required effort before response onset, while noradrenaline reflected the grip force actually exerted during responses, which also was reflected in pupil diameter. These findings suggest a link between noradrenaline, pupil dilation, and physical effort expenditure that is likely shared across species.

While several studies have reported a correlation between pupil diameter and activity of the locus coeruleus (Joshi & Gold, 2019; Joshi et al., 2016; Murphy, O’Connell, O’Sullivan, Robertson, & Balsters, 2014), this link has recently come under debate (Megemont, McBurney-Lin, & Yang, 2022). Pupil size also correlates with the trial-by-trial BOLD signal activity in other brain stem nuclei, specifically the dopaminergic ventral tegmental area and substantia nigra, at least during rest (Lloyd, de Voogd, Mäki-Marttunen, & Nieuwenhuis, 2023). It might be interesting to consider the possibility that the response-induced modulation of pupil dilation in this study in fact reflects dopaminergic activity (Varazzani et al., 2015; Walton & Bouret, 2018). In line with this hypothesis, one of our past studies (Algermissen et al., 2022) found the same pattern observed in pupil dilations in this study—a strong main effect of responses, with a particular strong signal for responses to Avoid cues—in the dorsal striatal BOLD signal, which replicated previous patterns of VTA and striatal BOLD signal (Guitart-Masip et al., 2012) and was recently replicated again (Queirazza et al., 2023). The same study found striatal BOLD to be correlated with midfrontal theta power. Other studies have found pupil diameter to be related to midfrontal theta power (Dippel et al., 2017; Lin et al., 2018) and the P300, an evoked potential likely generated by stimulus-locked oscillations in the theta range (de Gee, Correa, Weaver, Donner, & van Gaal, 2021; Murphy, Robertson, Balsters, & O’Connell, 2011; Nieuwenhuis, Aston-Jones, & Cohen, 2005). In sum, striatal BOLD, midfrontal theta power, and pupil diameter might all reflect the same underlying signal, which however is not noradrenergic, but dopaminergic in nature.

If pupil dilation indexes dopaminergic processes in the striatum, these might be directly related to the “unfreezing” of gaze. Striatal dopamine has been suggested to enhance the contrast of cortical action representations against background noise, facilitating their selection (Nicola, Woodward Hopf, & Hjelmstad, 2004). The early freezing evoked by activation of the indirect pathway, visible in reduced gaze dispersion, might be directly counteracted by the dopaminergic enhancement of action representations in the direct pathway, visible in pupil dilation, which would bridge the two main findings of this study. Interestingly, while freezing of gaze became stronger over time within a task block, potentially reflecting more automatic retrieval of the valence of Avoid cues, the incremental pupil dilation associated with Go responses to Avoid cues showed an inverted-U shaped time course, diminishing towards the end of task blocks. This observation tentatively suggests that counteracting the Pavlovian biases also becomes more automatized over time, requiring less physical effort with practice. Future research will have to use brain imaging methods to directly investigate the time course of such effort-related processes in the striatum.

The hypothesis that pupil dilation (at least partially) reflects dopaminergic processes in the striatum is in line with recent accounts of the role of dopamine in motivating action. Specifically, it has been proposed that the striatum evaluates whether recruiting additional effort to invigorate a candidate response option will lead to increases in expected reward, i.e., it computes the “value of work” (Hamid et al., 2016; Mohebi et al., 2019; Syed et al., 2016; Westbrook, Frank, & Cools, 2021). Further work by the same authors has suggested that dopamine reflects the control or “agency” an individual experiences over its environment, reflecting whether it is worth investing effort to try to increase reward rates (Hamid, 2021; Hamid, Frank, & Moore, 2021). The value of work is particularly high under response conflict, when boosting slow, controlled response options over fast, automatic, prepotent response options can make a difference for whether a correct response is executed (and a reward obtained). Hence, pupil dilation might give insights into this underexplored facet of cognitive control.

### Limitations

The present study features a number of limitations and points at new directions for future research. First, the unsuccessful subliminal manipulation motivates the question whether a supraliminal manipulation might be more successful. However, for supraliminally presented stimuli, even more care must be taken in matching their visual properties, and condition differences could reflect differences in low-level stimulus processing. Furthermore, consciously perceived emotional stimuli can induce high-level changes in response strategy, i.e., demand characteristics (Mahlberg et al., 2021), which necessitates the use of an elaborate and effective cover story. Lastly, the presence of strong response-related transients in the pupil data might potentially camouflage more subtle stimulus-induced effects. Other physiological measures of arousal, such as heart rate and skin conductance, might be more suitable to measure the effects of supraliminally presented arousing stimuli (Hashemi et al., 2019; Klaassen et al., 2021). However, these measures need much longer measurement periods, requiring a slower trial structure.

In the present data, pupil diameter peaked around 1,600 ms after stimulus onset and returned to baseline around 3,000 ms, showing a slower time course than previous studies on pupil dilation (Hoeks & Levelt, 1993) and warranting care when preregistering analysis windows. The time course of the pupil dilation might vary considerably as a function of the task structure and should be measured in pilot data before preregistering a definite analysis window.

### Conclusions

Our results shed new light on the effects of aversive cues on motor behavior (eye and hand movements) and on the effortful counter-mechanisms recruited to overcome aversive inhibition. Aversive cues reduced response rates, slowed responses and reduced gaze dispersion (“freezing of gaze”). Over time, participants learned to counteract these aversive Pavlovian biases and make Go responses even to aversive cues. These responses were associated with particularly strong and sustained pupil dilations, which we interpret as reflecting additional physical effort recruitment in order to overcome aversive inhibition. While previous literature has primarily focused on how impulsive responding to Win cues can be suppressed (Cavanagh et al., 2013; Swart et al., 2018), this study sheds light on the opposite end of Pavlovian biases, namely how humans can invigorate responding against factors holding them back. Future studies could use pupillometry in the context of aversive inhibition to further probe this underexplored facet of cognitive control.

### Supplementary Information

Below is the link to the electronic supplementary material.

Supplementary file1 (PDF 3.30 MB)

## Data Availability

All code, raw data and pre-processed data required to reproduce the reported results are available under: 10.34973/vh63-k490. Code will be maintained under https://github.com/johalgermissen/Algermissen2024CABN, with a permanent copy at the time of publication under https://github.com/denoudenlab/Algermissen2024CABN.

## Declarations

### Previous dissemination

A preprint of this work has been posted under https://www.biorxiv.org/content/10.1101/2023.12.28.573353v2.
