# Supplementary material for: Pupil dilation reflects effortful action invigoration in overcoming aversive Pavlovian biases
Source: Cogn Affect Behav Neurosci. 2024 May 21;24(4):720–39. doi: 10.3758/s13415-024-01191-y (PMC11233311; doi:10.3758/s13415-024-01191-y)
Supplement: Supplementary file 1 — Supplementary file1 (PDF 3.30 MB) [file 13415_2024_1191_MOESM1_ESM.pdf]

Supplementary Files

Table of Contents

Supplementary Material S01: Overview of results from all mixed-effects regression models2

Supplementary Material S02: Overview of means and standard deviations of responses and RTs per task condition ... 4

Supplementary Material S03: Effect of response accuracy and cue valence on RTs over time ... 5

Supplementary Material S04: Correlations of the effects of cue valence on responses and RTs with questionnaires ... 7

Supplementary Material S05: No effect of arousal manipulation on the responses, RTs, and pupil dilation ... 8

Supplementary Material S06: Association of pupil dilation with responses and RTs... 13

Supplementary Material S07: Higher pupil dilations for responses to Avoid cues than to Win cues while controlling for accuracy, RTs, and response repetition over time ... 18

Supplementary Material S08: Association of pupil baseline with accuracy, RTs, and response repetition over time ... 24

Supplementary Material S09: Outcome-locked pupil dilation ... 29

Supplementary References ... 32

Supplementary Material S01: Overview of results from all mixed-effects regression models

Here, we report an overview over all major statistical results reported in the main text and the Supplementary Materials S05 and S06. For details on how mixed-effects regression were performed, see the Methods section of the main text.

Model ID	DV	IV	*b*	*SE*	$\chi^2(1)$	*p*
1	Response	Req. action	1.367	0.096	66.423	< .001
		Valence	0.537	0.100	20.986	< .001
		Req. action x valence	0.068	0.057	1.238	.246
2	RT	Req. action	-0.143	0.028	20.446	< .001
		Valence	-0.161	0.025	27.329	< .001
		Req. action x valence	-0.007	0.023	0.083	.773
3	Response	Req. action	1.368	0.097	66.422	< .001
		Valence	0.539	0.101	20.957	< .001
		Manipulation	-0.008	0.028	0.054	.816
		Req. action x valence	0.068	0.058	1.321	.250
		Req. action x manipulation	-0.019	0.028	0.319	.573
		Valence x manipulation	0.006	0.030	0.034	.854
		Req. action x valence x manipulation	-0.014	0.029	0.170	.680
4	RT	Req. action	-0.141	0.028	26.046	< .001
		Valence	-0.159	0.025	40.344	< .001
		Manipulation	-0.005	0.017	0.080	.777
		Req. action x valence	-0.009	0.023	0.152	.697
		Req. action x manipulation	0.014	0.017	0.713	.398
		Valence x manipulation	0.008	0.018	0.211	.646
		Req. action x valence x manipulation	-0.025	0.016	2.477	.116
5	Response	Req. action	1.379	0.096	67.271	< .001
		Valence	0.560	0.101	21.971	< .001
		Dilation	0.309	0.054	22.519	< .001
		Req. action x valence	0.091	0.059	2.246	.134
		Req. action x dilation	-0.119	0.036	7.945	.005
		Valence x dilation	-0.004	0.041	0.009	.924
		Req. action x valence x dilation	-0.012	0.042	0.065	.799
6	RT	Req. action	-0.144	0.027	21.532	< .001
		Valence	-0.146	0.025	23.429	< .001
		Dilation	0.096	0.017	43.879	< .001
		Req. action x valence	-0.013	0.023	0.305	.580
		Req. action x dilation	0.039	0.017	5.338	.021
		Valence x dilation	-0.034	0.018	3.140	.076
		Req. action x valence x dilation	0.004	0.017	0.057	.812
7	Response	Req. action	1.386	0.096	67.406	< .001
		Valence	0.563	0.101	22.201	< .001
		Manipulation	0.013	0.030	0.154	.695d
		Dilation	0.327	0.053	25.649	< .001
		Req. action x valence	0.090	0.059	2.121	.145
		Req. action x manipulation	-0.014	0.031	0.123	.726
		Valence x manipulation	0.018	0.031	0.259	.611
		Req. action x dilation	-0.109	0.038	5.907	.015
		valence x dilation	-0.003	0.042	0.021	.886
		Manipulation x dilation	0.024	0.033	0.370	.543
		Req. action x valence x manipulation	-0.011	0.032	0.087	.768
		Req. action x valence x dilation	-0.001	0.044	0.020	.887
		Req. action x manipulation x dilation	0.023	0.033	0.360	.549
		Valence x manipulation x dilation	0.001	0.033	0.019	.891
		Req. action x valence x manipulation x dilation	0.027	0.036	0.420	.517
8	RT	Req. action	-0.145	0.027	22.266	< .001
		Valence	-0.146	0.025	24.679	< .001

	Manipulation	-0.008	0.017	0.230	.631
	Dilation	0.093	0.018	19.654	< .001
	Req. action x valence	-0.012	0.023	0.287	.592
	Req. action x manipulation	0.018	0.017	0.998	.318
	valence x manipulation	0.010	0.017	0.316	.574
	Req. action x dilation	0.041	0.017	5.476	.019
	valence x dilation	-0.033	0.018	2.979	.084
	Manipulation x dilation	0.011	0.016	0.509	.475
	Req. action x valence x manipulation	-0.032	0.016	3.661	.056
	Req. action x valence x dilation	0.003	0.017	0.019	.891
	Req. action x manipulation x dilation	-0.024	0.017	1.867	.172
	Valence x manipulation x dilation	-0.031	0.019	2.452	.117
	Req. action x valence x manipulation x dilation	0.024	0.016	3.1817	.051

Table S01. Overview of the results from all mixed-effects logistic and linear regression models reported in the main text of the manuscript.

Supplementary Material S02: Overview of means and standard deviations of responses and RTs per task condition

Responses

Req. Act.	Go	Go	NoGo	NoGo
Valence	Win	Avoid	Win	Avoid
Mean	0.875	0.759	0.410	0.216
SD	0.124	0.122	0.258	0.096

Table S02. Means and standard deviations of Go/NoGo responses across participants per required action x valence condition.

Responses

Req. Act.	Go	Go	Go	Go	NoGo	NoGo	NoGo	NoGo
Valence	Win	Win	Avoid	Avoid	Win	Win	Avoid	Avoid
Prime	High	Low	High	Low	High	Low	High	Low
Mean	0.871	0.880	0.754	0.763	0.414	0.405	0.215	0.217
SD	0.131	0.124	0.138	0.124	0.258	0.269	0.106	0.102

Table S03. Means and standard deviations of Go/NoGo responses across participants per required action x valence x prime condition.

RTs

Req. Act.	Go	Go	NoGo	NoGo
Valence	Win	Avoid	Win	Avoid
Mean	0.641	0.707	0.707	0.756
SD	0.071	0.076	0.122	0.103

Table S04. Means and standard deviations of reaction times across participants per required action x valence condition.

RTs

Req. Act.	Go	Go	Go	Go	NoGo	NoGo	NoGo	NoGo
Valence	Win	Win	Avoid	Avoid	Win	Win	Avoid	Avoid
Prime	High	Low	High	Low	High	Low	High	Low
Mean	0.641	0.641	0.713	0.702	0.711	0.704	0.738	0.771
SD	0.081	0.067	0.078	0.083	0.131	0.123	0.131	0.116

Table S05. Means and standard deviations of reaction times across participants per required action x valence x prime condition.

Supplementary Material S03: Effect of response accuracy and cue valence on RTs over time

In the main text, we report main effects of accuracy/ required action on RTs, with slower RTs for incorrect responses (to NoGo cues) than for correct responses (to Go cues), and of cue valence on RTs, with slower responses to Avoid cues than to Win cues. Here, we fitted additional generalized additive mixed-effects models to test whether these effects changed over trials (i.e., cue repetitions).

For the time course of RTs across trials per condition, see Fig. S01A. Again, we found significantly slower RTs for incorrect responses (to NoGo cues) than for correct responses (to Go cues) for cue repetitions 3–13, parametric term $t(3.19, 0.11) = 3.726$, $p < .001$, smooth term $F(3.03, 3.73) = 2.894$, $p = .023$ (Fig. S01B). The fact that incorrect responses were significantly slower already on the third repetition of a cue reveals that participants had some (partial) awareness of the correct response already after the first few trials. Differences disappeared towards the end of blocks. Note that accuracy increased over time, with fewer and fewer trials contributing to the time courses of incorrect responses. Changes in the number of trials contributing to conditions likely explain why the disappearance of this effect seems to be at odds with FigS01A, in which the time course for incorrect responses to Avoid cues (red dashed line) continues to become slower. Accuracy for NoGo-to-Avoid trials is very high, especially late in the blocks (see Fig. 2A in the main text), with mostly NoGo responses in this condition and very few incorrect Go responses that contribute to the RT time course. These few incorrect NoGo-to-Avoid trials are down-weighted relative to the more frequent incorrect NoGo-to-Win trials.

Furthermore, we found significantly slower RTs for responses to Avoid cues than for responses to Win cues for cue repetitions 2–16, parametric term $t(3.36, 0.11) = 12.851$, $p < .001$, smooth term $F(3.31, 4.06) = 8.790$, $p < .001$(Fig. S01C). Pavlovian biases in RTs emerged already on the 2nd repetition of a cue and continued until the end of blocks.

In sum, these results reflect that effects of required action/ accuracy and cue valence on RTs emerged after the first few trial repetitions. While the accuracy effect disappeared towards the end of blocks (with few incorrect Go responses to NoGo cues), the cue valence effect persisted till the end.

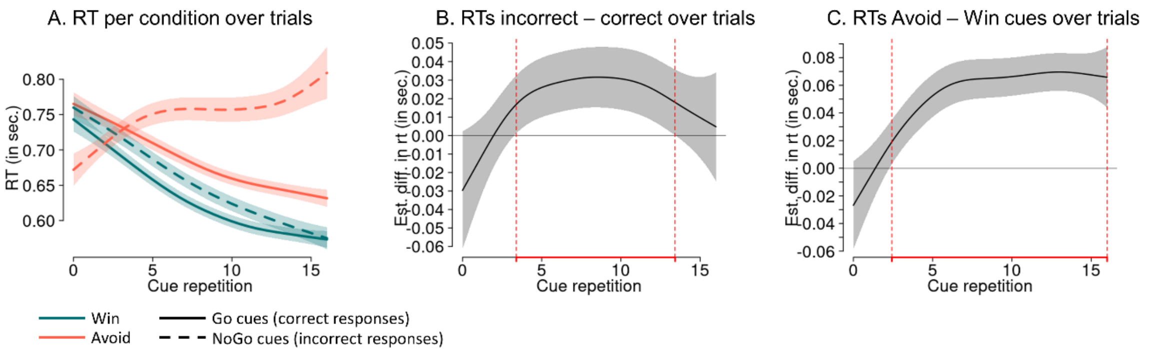

Figure S01. Effect of accuracy and cue valence on RTs over cue repetitions. **A.** Time course of dilations over cue repetitions (mean ± SE) as predicted from a generalized additive mixed-effects model (GAMM), separated by accuracy/ required action and cue valence. RTs are significantly slower for incorrect than correct responses from cue repetition 3 to 13 and significantly slower for responses to Avoid than Win cues from cue repetition 2 to 16 (i.e., the end of blocks). **B.** Difference line between RTs for incorrect responses minus correct responses, with significant differences from cue repetition 3 to 13. Areas highlighted in red indicate time windows with significant differences. **C.** Difference line between RTs for responses Avoid cues minus Win cues, with significant differences from cue repetition 2 to 16.

Supplementary Material S04: Correlations of the effects of cue valence on responses and RTs with questionnaires

In line with the exploratory analysis plans in mentioned in our pre-registration, we extracted the per-participant coefficients (fixed plus random effects) for (a) the effect of cue valence on responses and RTs (Pavlovian bias), (b) the effect of the arousal manipulation on responses and RTs, and (c) the effect of pupil dilation on responses and RTs. We then computed correlations of these coefficients with trait anxiety (STAI, Form Y-2, 20 items) (Spielberger, Gorssuch, Lushene, Vagg, & Jacobs, 1983) and the five sub-scales negative urgency, lack of perseveration, lack of premeditation, sensation seeking, and positive urgency of the UPPS-P Impulsive Behavior Scale (short version, 20 items) (Cyders, Littlefield, Coffey, & Karyadi, 2014). One might plausibly hypothesize that impulsivity is related to the Pavlovian bias since many impulsive behaviors can be conceptualized as automatic, cue-triggered behaviors.

See Fig. S02 for scatterplots of all bivariate associations. None of these correlations were significant at $\alpha = .05$ (uncorrected), providing no evidence for the strength of the Pavlovian bias in responses or RTs being related to either trait anxiety or sub-facets of impulsivity. Note that these analysis are underpowered to detect correlations of small-to-moderate size: With $N = 35$, we have 80% power to detect correlations of $|r| > 0.45$, and only correlations of $|r| > 0.33$ (50% power) will become significant.

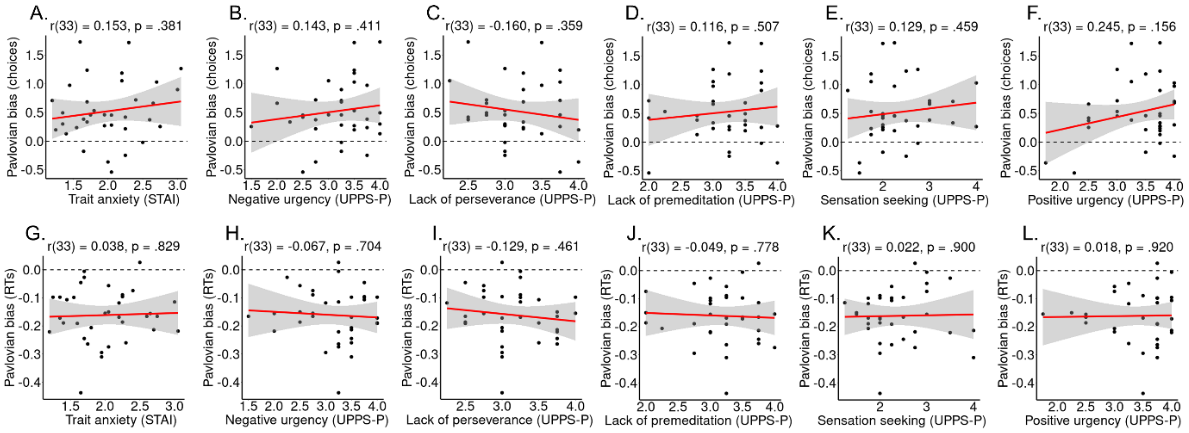

Figure S02. Association of trait anxiety and various sub-facets of trait impulsivity with the effect of valence on responses and RTs. Correlations between the effect of valence on responses (**A–F**) and on RTs (**G-L**), reflecting Pavlovian biases, and trait anxiety (**A**, **G**), negative urgency (**B, H**), lack of perseverance (**C, I**), lack of premeditation (**D, J**), sensation seeking (**E, K**), and positive urgency (**F, L**). Black dots represent per-participant scores, the red line the best-fitting regression line, the grey shade the 95%-confidence interval. None of the displayed correlations is significant at α = .05.

Supplementary Material S05: No effect of arousal manipulation on the responses, RTs, and pupil dilation

Here, we report the full results from confirmatory pre-registered and additional exploratory analyses of a responses, RTs, and trial-by-trial pupil dilation as function of the subliminal arousal manipulation (angry vs. neutral face primes). In brief, there was no effect of the arousal manipulation on responses, RTs, or pupil dilation, neither as a main effect nor in interaction with other task factors or measured pupil dilation. In pre-registered, confirmatory models, we fit a mixed-effects logistic regression model with responses (Go/NoGo) as dependent variable and required action (Go/ NoGo), cue valence (Win/ Avoid), arousal priming manipulation (angry/ neutral face), as well as all possible interactions between them as independent variables. Furthermore, we fit an equivalent exploratory linear regression model with RTs as dependent variable. As a initial, exploratory manipulation check, we fit an equivalent linear regression model with trial-by-trial pupil dilation as dependent variable.

As a initial manipulation check, we performed exploratory analyses testing for an effect of the subliminal arousal manipulation (angry vs. neural face primes) on trial-by-trial-pupil dilation. We expected higher pupil dilations for angry compared to neural faces, reflecting heightened arousal induced by angry faces. In a mixed-effects model regression trial-by-trial pupil dilations onto the conditions of the arousal manipulation, there was no effect of the manipulation, b = -0.003, 95%-CI [-0.022, 0.017], $\chi^2(1) = 0.071$, p = .790 (Fig. S03A), providing no evidence for the subliminal arousal manipulation affecting arousal as index by pupil diameter. In addition to this regression model, we performed two more exploratory manipulation checks, testing for differences between subliminally presented angry vs. neutral faces at any time point during a trial as well as at any time point during learning.

To test for any effect of the arousal manipulation on pupil dilation at any time point within a trial, we computed the raw pupil time course per condition (high vs. low arousal) for every participant and then the average per condition across participants. A cluster-based permutation test yielded no significant difference at any time point (no cluster above the cluster-forming threshold of $|t| > 2$), suggesting again no effect of the arousal manipulation on pupil dilation (Fig. S03B).

Furthermore, we tested whether the arousal manipulation affected pupil dilations at any time point within a block using generalized additive mixed-effects models. There was no difference in the trial-by-trial time course of pupil dilations between high-arousal and low-arousal trials, linear term $t(5.75, 7.61) = 0.252$, p = .801, smooth term $F(2.42, 2.98) = 1.757$, $p = .170$, suggesting again no effect of the arousal manipulation on pupil dilation (Fig. S03C). In sum, none of the performed manipulation checks suggested any evidence for the subliminal arousal manipulation affecting arousal as indexed by trial-by-trial fluctuations in pupil dilations.

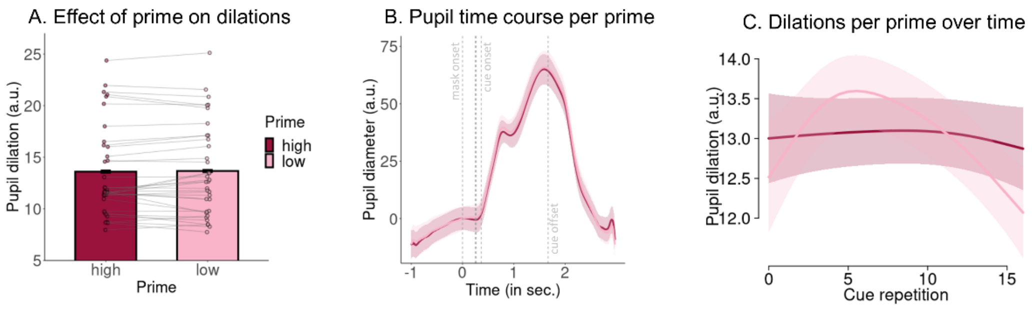

Figure S03. Effect of arousal manipulation on pupil dilation. **A.** Mean pupil dilation per level of the arousal priming manipulation (whiskers are ±SEM across participants, dots indicate individual participants). There is no effect of the arousal priming manipulation on pupil dilations. **B.** Pupil time course within a trial (mean ± SE; baseline-corrected) separately for high vs. low arousal condition. Vertical dashed lines indicate the onset of the forward mask (at 0 ms), the prime (at 250 ms), the backwards mask (at 266 ms), the cue onset (at 366 ms), and the cue offset (at 1666 ms). There is no significant difference (no cluster above cluster-forming threshold). **C.** Time course of dilations over cue repetitions (mean ± SE) as predicted from a generalized additive mixed-effects model (GAMM), separated by arousal condition. There is no significant difference in pupil dilation between conditions at any time point.

Next, we proceed with our main confirmatory, pre-registered analyses. As a first set of confirmatory, pre-registered analyses, we extended the regression model in the main text fitting responses as a function of required action and cue valence by adding the arousal priming manipulation (high/ low, i.e., angry/ neutral face stimulus) and all higher-order interactions possible. Neither the main effect of the arousal priming manipulation, $b = -0.008$, 95%-CI [-0.063, 0.047], $\chi^2(1) = 0.054$, $p = .816$, nor the 2-way interaction between the priming manipulation and cue valence, $b = 0.006$, 95%-CI [-0.052, 0.065], $\chi^2(1) = 0.034$, $p = .854$, nor the 3-way interaction between the priming manipulation, cue valence, and required action, $b = -0.014$, 95%-CI [-0.071, 0.043], $\chi^2(1) = 0.170$, $p = .680$, was significant, providing no evidence for any effect of the priming manipulation on responses (Fig. S04A-C).

Fitting an equivalent model to RTs, neither the main effect of the arousal priming manipulation, $b = -0.005$, 95%-CI [-0.038, 0.028], $\chi^2(1) = 0.073$, $p = .787$, nor the 2-way interaction between the priming manipulation and cue valence, $b = 0.008$, 95%-CI [-0.026, 0.043], $\chi^2(1) = 0.197$, $p = .657$, nor the 3-way interaction between the priming manipulation, cue valence, and required action, b = -0.025,

95%-CI [-0.055, 0.006], $\chi^2(1) = 2.354$, $p = .125$, was significant, providing no evidence for any effect of the arousal priming manipulation on responses (Fig. S04D-F). Taken together, none of the confirmatory analyses provided any evidence for the arousal priming manipulation affecting behavior.

As a third set of confirmatory analyses, we fit a regression model with required action, cue valence, the arousal priming manipulation, trial-by-trial pupil dilation, and all higher-order interactions possible. There was no significant 4-way interaction on either responses, $b = 0.027$, 95%-CI [-0.044, 0.098], $\chi^2(1) = 0.420$, $p = .517$, nor RTs, b = 0.024, 95%-CI [-0.006, 0.055], $\chi^2(1) = 3.817$, $p = .051$, again providing no evidence for an effect of the arousal priming manipulation, also not as a function of the trial-by-trial pupil dilation.

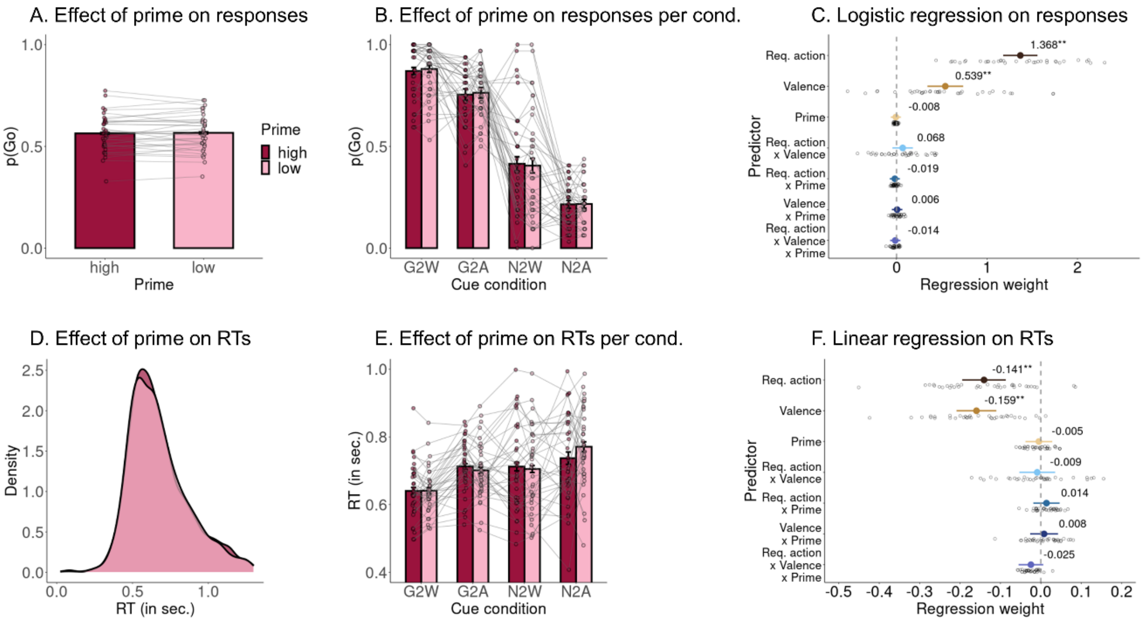

Figure S04. Effect of the arousal priming manipulation on responses and RTs. **A.** Proportion of Go responses for high (angry face) and low arousal (neutral face) priming manipulation (whiskers are ± SEM across participants, dots indicate individual participants). There is no effect of the manipulation on responses. **B**. Proportion of Go responses for high and low arousal priming manipulation separately per cue condition. There is no effect of the manipulation on responses for any condition. **C.** Group-level (colored dot, 95%-CI) and individual-participant (grey dots) regression coefficients from a mixed-effects logistic regression of responses on required action, cue valence, the arousal priming manipulation, and all higher-order interactions. None of the terms involving the arousal priming manipulation is significant. **D**. Distribution of raw RTs separately per arousal priming manipulation level. There is no difference between both levels. **E**. Mean RTs for high and low arousal priming manipulation separately per cue condition. There is no effect of the manipulation on RTs for any condition. **F.** Group-level and individual-participant regression coefficients from a mixed-effects linear regression of RTs on required action, cue valence, the arousal priming manipulation, and all higher-order interactions. None of the terms involving the arousal priming manipulation is significant.

As an exploratory check, we tested whether individual differences in the effects of the arousal manipulation on responses, RTs, and pupil dilation were correlated, i.e., whether only those participants who showed an effect on pupil dilation also showed an effect on behavior. For this purpose, we fit regression models with the manipulation as sole independent variable and responses, RTs, and dilations and dependent variables, extracted the per-participants coefficients (fixed + random effects), and

correlated them. Neither the per-participants effects of the manipulation on dilations and responses, $r(33) = -0.202$, $p = .243$ (Fig. S05A), nor the effects on dilations and RTs, $r(33) = 0.121$, $p = .487$ (Fig. S05B), were significantly correlated, providing no evidence for systematic individual differences in the effect of the arousal manipulation on behavior and physiology.

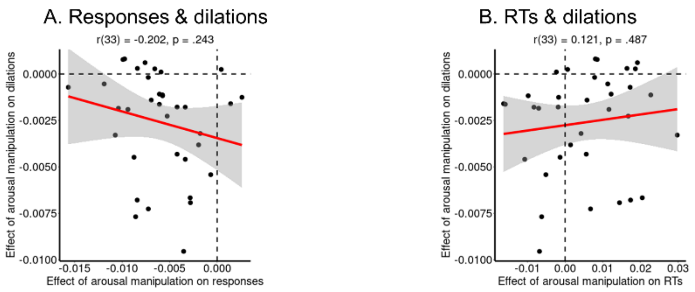

Figure S05. Correlations between the effects of the arousal manipulation on response, RTs, and pupil dilations. **A.** Correlation between the effect of the arousal manipulation on responses and on trial-by-trial pupil dilation. Black dots represent per-participant scores, the red line the best-fitting regression line, the grey shade the 95%-confidence interval. The correlation is not significant. **B**. Correlation between the effect of the arousal manipulation on RTs and on trial-by-trial pupil dilation. The correlation is not significant.

As a final set of exploratory analyses, we tested whether individual differences in the effect of the arousal priming manipulation on responses or RTs were correlated with individual differences in self-reported anxiety and/or impulsivity. One might plausibly hypothesize that trait anxiety would be associated with a stronger effect of the exogenously induced arousal on responses and RTs. See Fig. S06 for scatterplots of all bivariate associations. Neither the effect of the arousal priming manipulation on responses nor on RTs was correlated with trait anxiety or impulsivity across participants, providing no evidence for the strength of the effect of induced arousal on responses and RTs being related to either trait anxiety or sub-facets of impulsivity. Note that these analysis are underpowered to detect correlations of small-to-moderate size: With $N = 35$, we have 80% power to detect correlations of $|r| > 0.45$, and only correlations of $|r| > 0.33$ (50% power) will become significant.

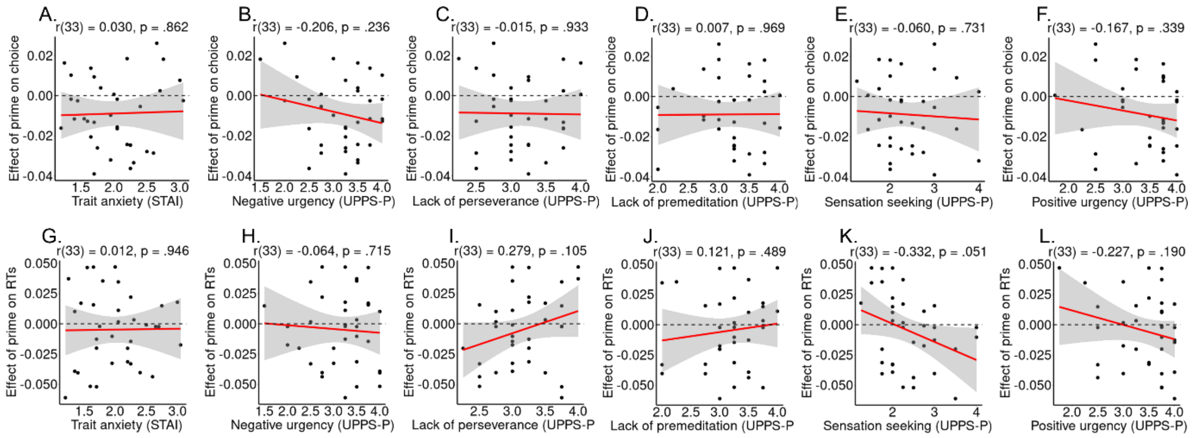

Figure S06. Association of trait anxiety and various sub-facets of trait impulsivity with the effect of the arousal manipulation on responses on RTs. Correlations between the effect of the subliminal arousal manipulation on responses (**A–F**) and on RTs (**G-L**), and trait anxiety (**A**, **G**), negative urgency (**B, H**), lack of perseverance (**C, I**), lack of premeditation (**D, J**), sensation seeking (**E, K**), and positive urgency (**F, L**). Black dots represent per-participant scores, the red line the best-fitting regression line, the grey shade the 95%-confidence interval. None of the displayed correlations is significant at α = .05.

In this study, we used a previously established manipulation that subliminally presented faces with angry or neutral faces to induce high vs. low arousal (Allen et al., 2016). We did not observe any effects on responses, RTs, or pupil dilation. Confidence intervals and raw data plots indicated that the effect of the manipulation on all dependent measures was close to zero (Fig. S04), with little variation across participants, providing strong evidence for a null effect. Hence, although this procedure has been used successfully in the past (and proven seemingly effective in data from four pilot participants we had collected initially), it was unsuccessful in this study. Likely, the presentation duration was too short for participants to (even subliminally) process the emotional faces. The pupillometry data in particular provides strong evidence that no processing of the emotional faces occurred. This failure to use a subliminal manipulation to induce arousal aligns with other recent reports calling into question the effectiveness of subliminal manipulations reported in the literature (Mudrik & Deouell, 2022). Several cognitive processes previously reported to occur without awareness, including emotional face processing, might in fact require awareness (Mudrik & Deouell, 2022; Skora, Livermore, Dienes, Seth, & Scott, 2023; Vadillo, Malejka, Lee, Dienes, & Shanks, 2022). It is possible that subsets of participants who perceived stimuli supraliminally did in fact drive seemingly subliminal effects in past studies (Skora et al., 2023).

Supplementary Material S06: Association of pupil dilation with responses and RTs

In the main text of the manuscript, we report exploratory regression models with pupil dilations as dependent variable and task factors such as the performed response or cue valence as independent variables. However, a complementary approach, which we in fact mentioned in our pre-registration, are regression models with dependent and independent variables inverted, i.e., using responses and RTs as dependent variables and trial-by-trial fluctuations in pupil dilation as independent variable, including interactions between pupil dilation and the task factors cue valence and required action.

Note that these analyses are harder to interpret with regard to cognitive effort and physical effort accounts of pupil dilation since they do not directly contrast the relevant task conditions. Nonetheless, the results from these analyses are largely in line with the results reported in main text, with high pupil dilations being associated with Go responses and with particularly with slower RTs.

As confirmatory models, we fit a mixed-effects logistic regression model with responses (Go/NoGo) as dependent variable and required action (Go/ NoGo), cue valence (Win/ Avoid), trial-by-trial pupil dilations, as well as all possible interactions between them as independent variables. Furthermore, we checked whether including RTs as an additional independent variable or including the interaction between RTs and trial-by-trial pupil dilation as an additional independent variable led to different results. Adding those additional independent variables lead to identical conclusions. These models are thus not separately reported here. In case of interactions between dilations and task conditions, in order to better understand these effects, we combined required action and cue valence into a single "cue condition" variable and fit a model with dilation, cue condition, and their interaction. We then tested for differences between conditions in the slope of the dilation effect using z-tests provided by the emmeans package in R, which corrects for multiple comparisons using the Tukey method.

In these analyses, we extended the regression model reported in the main text fitting responses as a function of required action and cue valence by adding the trial-by-trial pupil dilation and all possible higher-order interactions. There was a significant main effect of dilation, b = 0.309, 95%-CI [0.203,

0.414], $\chi^2(1) = 22.519$, $p < .001$, with overall stronger dilations for Go responses (Fig. S07A, C). Furthermore, there was a significant interaction between dilations and required action, $b = -0.119$, 95%-CI [-0.19, -0.049], with a stronger relationship for incorrect responses (to NoGo cues) than correct responses (to Go cues) (Fig. S07B, C). In contrast, neither the 2-way interaction between dilations and cue valence $b = -0.004$, 95%-CI [-0.085, 0.077], $\chi^2(1) = 0.009$, $p = .923$, nor the 3-way interaction between dilations, cue valence, and required action was significant, $b = -0.012$, 95%-CI [-0.095, 0.071], $\chi^2(1) = 0.065$, $p = .799$, providing no evidence for pupil dilation modulating the effect of Pavlovian biases on responses (Fig. S07C). To better understand the 2-way interaction between dilations and required action, we fit a follow-up model combining required action and cue valence into a single "cue condition" variable with 4 levels (Go-to-Win, Go-to-Avoid, NoGo-to-Win, NoGo-to-Avoid). The 2-way interaction between dilations and conditions was significant, $\chi^2(1) = 8.977$, $p = .030$. The association between dilation and the probability of making a Go response was positive in all conditions, with a marginally significant tendency for a stronger link for NoGo-to-Win cues than for Go-to-Win cues ($z = 2.409$, $p = 0.076$) and for Go-to-Avoid cues ($z = 2.406$, $p = .076$), but overall no significant difference between pairs of conditions. See Supplementary Material S07 for evidence that the stronger dilations for incorrect responses (to NoGo cues) than correct responses (to Go cues) occurred due to the former being overall slower, with the association between pupil dilations and accuracy vanishing when controlling for RT differences. In sum, Go responses were associated with stronger pupil dilation, which was especially the case for responses to NoGo cues (i.e., when those responses were incorrect and slow), but there was no evidence for dilations modulating the Pavlovian bias in responses.

An equivalent model fit to RTs yielded a significant main effect pupil dilation, $b = 0.096$, 95%-CI [0.062, 0.129], $\chi^2(1) = 43.879$, $p < .001$, with stronger dilations being associated with slower RTs, and a significant 2-way interaction between dilations and required action, $b = 0.039$, 95%-CI [0.007, 0.072], $\chi^2(1) = 5.338$, $p = .021$, with a stronger link between dilations and RTs for Go cues compared to NoGo cues (Fig. S07E, F). The 2-way interaction between dilations and cue valence was only marginally significant, $b = -0.034$, 95%-CI [-0.070, 0.002], $\chi^2(1) = 3.140$, $p = .076$, tending towards a stronger link between dilations and RTs for Avoid compared to Win cues (Fig. S07D, F). The 3-way interaction

between dilations, cue valence, and required action was no significant, $b = 0.004$, 95%-CI [-0.03, 0.038], $\chi^2(1) = 0.057$, $p = .812$ (Fig. S07F).

To better understand the (marginally) significant 2-way interactions, i.e., test explicitly whether effects were driven by only one of the four cue conditions, we again fit a follow-up model combining required action and cue valence into a single "cue condition" variable with four levels. The 2-way interaction between dilation and cue condition was significant, $\chi^2(1) = 9.603$, $p = .023$. The association between dilations and RTs was positive in all conditions, strongest in the Go-to-Avoid condition, and weakest in the NoGo-to-Win condition, with this difference being significant, $z = 3.339$, $p = .005$, but none of the other comparisons being significant $p > .108$. See Supplementary Material S07 for evidence that the association between strong pupil dilations and slow RTs also explains the association between pupil dilations and incorrect responses. In sum, stronger dilations were associated with slower RTs, especially so for Go cues and Avoid cues, exacerbating the Pavlovian bias in RTs.

In sum, pupil dilations were stronger for Go responses, particularly for slow and for incorrect responses. The link between pupil dilation and RTs was stronger for Avoid compared to Win cues. However, this effect was only marginally significant and appeared to be driven by responses to Go-to-Avoid (rather than NoGo-to-Avoid) cues (though note that, for the latter, Go responses were incorrect, and thus only few trials with RTs were available). Taken together, these results are in line with a physical effort account of pupil dilation, with stronger dilations for Go than NoGo responses, overall, and particularly strong dilations in situations that require particular physical effort, such as responses to Avoid cues, (rare) incorrect responses, and particularly slow responses.

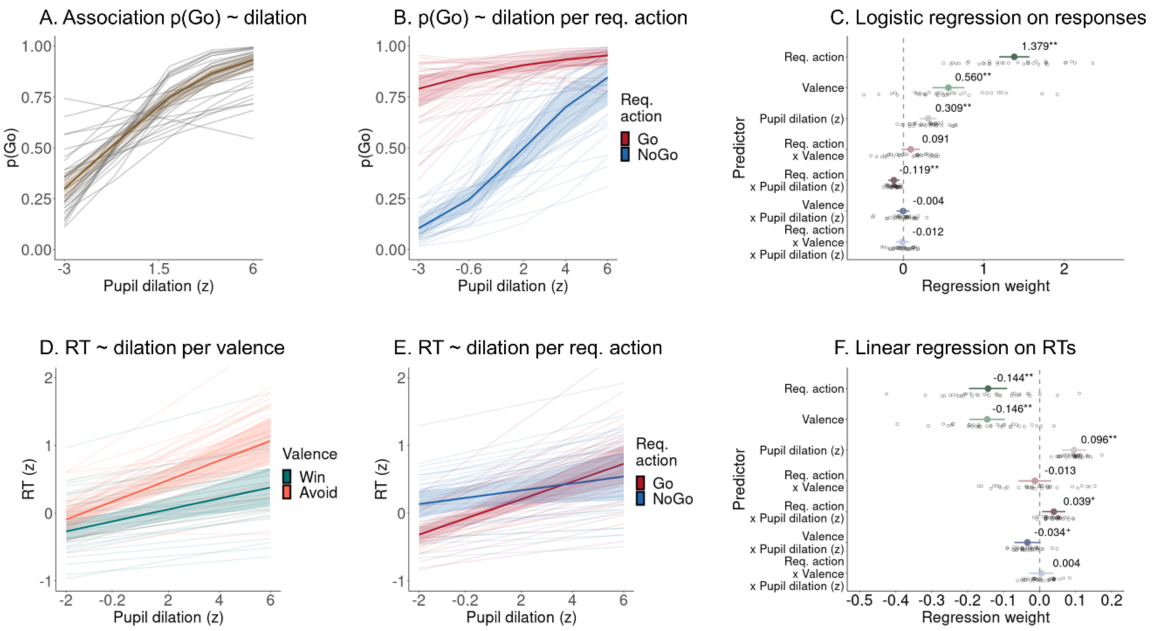

Figure S07. Effect of the trial-by-trial pupil dilation on responses and RTs. **A.** Proportion of Go responses as a function of trial-by-trial pupil dilation as predicted from a mixed-effects logistic regression model (colored line and shades are the group-level association + 95%-CIs; individual lines are the predictions for each individual participant). Go responses are associated with stronger pupil dilations. **B**. Predictions from panel A split per required action. The association between responses and pupil dilations is significantly stronger for (incorrect) responses to NoGo cues than for (correct) responses to Go cues. This difference between incorrect and correct responses is likely due to the former being slower than the latter (see Supplementary Material S07). **C.** Group-level (colored dot, 95%-CI) and individual-participant (grey dots) regression coefficients from a mixed-effects logistic regression of responses on required action, cue valence, pupil dilation, and all higher-order interactions. The main effect of pupil dilation and its interaction with required action are significant. **D**. Predictions of RTs from a mixed-effects logistic regression model based on trial-by-trial pupil dilation separately for Win and Avoid cues. Stronger pupil dilations are associated with slower responses. This relationship is marginally significantly stronger for Avoid than for Win cues. **E**. Predictions of RTs from a mixed-effects logistic regression model based on trial-by-trial pupil dilation separately for Go and NoGo cues. The association between pupil dilation and RTs is significantly stronger for (correct) responses to Go cues than (incorrect) responses to NoGo cues. **F.** Group-level and individual-participant regression coefficients from a mixed-effects linear regression of RTs on required action, cue valence, pupil dilation, and all higher-order interactions. The main effect of pupil dilation as well as its interaction with required action is significant; its interaction with cue valence is marginally significant.

As a final set of exploratory analyses, we tested whether individual differences in the link between pupil dilation and responses or RTs were correlated with individual differences in self-reported anxiety and/or impulsivity. One might plausibly hypothesize that trait anxiety would be associated with a stronger effect of endogenous arousal fluctuations as reflected in trial-by-trial pupil diameter on responses and RTs. See Fig. S08 for scatterplots of all bivariate associations. The only correlation significant at a level of $\alpha = .05$ (uncorrected) was between trait anxiety and the effect of dilations on RTs, with more anxious individuals showing a weaker link between trial-by-trial pupil dilation (supposedly reflecting fluctuations in endogenous arousal) and RTs. None of the other correlations were significant, providing no evidence for the strength of the effect of endogenous arousal on responses and RTs being related to either trait anxiety or sub-facets of impulsivity. Note that these analysis are underpowered to detect correlations of small-to-moderate size: With $N = 35$, we have 80% power to detect correlations of $|r| > 0.45$, and only correlations of $|r| > 0.33$ (50% power) will become significant.

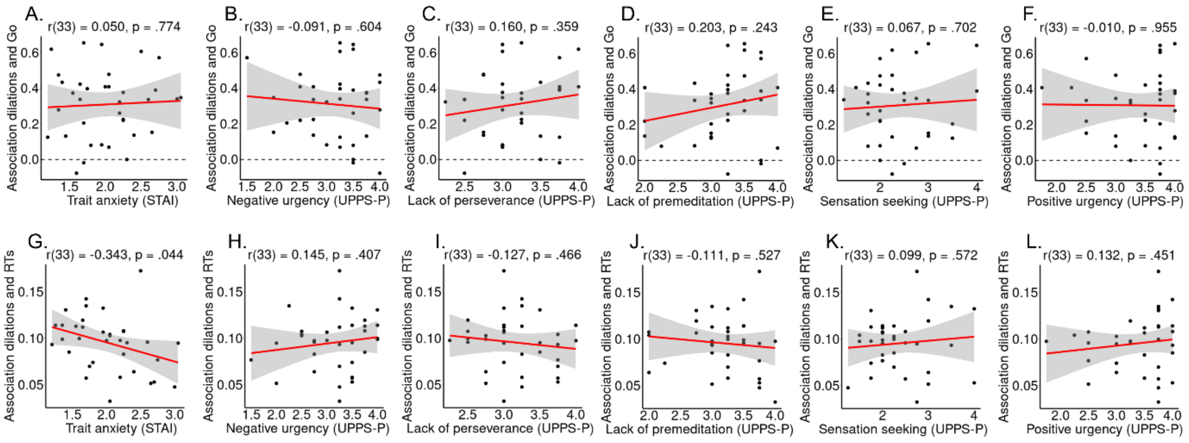

Figure S08. Association of trait anxiety and various sub-facets of trait impulsivity with the effect of trial-by-trial pupil dilation on responses on RTs. Correlations between the effect of trial-by-trial pupil dilation on responses (**A–F**) and on RTs (**G-L**), and trait anxiety (**A**, **G**), negative urgency (**B, H**), lack of perseverance (**C, I**), lack of premeditation (**D, J**), sensation seeking (**E, K**), and positive urgency (**F, L**). Black dots represent per-participant scores, the red line the best-fitting regression line, the grey shade the 95%-confidence interval. The only correlation significant at a level of $\alpha = .05$ (uncorrected) is between trait anxiety and the effect of dilations on RTs, with more anxious individuals showing a weaker link between trial-by-trial pupil dilation (supposedly reflecting fluctuations in endogenous arousal) and RTs.

Supplementary Material S07: Higher pupil dilations for responses to Avoid cues than to Win cues while controlling for accuracy, RTs, and response repetition over time

We performed control analyses testing whether the difference in pupil dilation between Go responses to Avoid compared to Win cues could be due to other factors associated with increased pupil dilations, specifically (a) correct vs. incorrect responses, (b) fast vs. slow responses (median split), and (c) response repetitions vs. switches to the alternative response option (with respect to the last encounter of the same cue).

See Table S06 for inferential statistics from mixed-effects linear regression models regressing trial-by-trial pupil dilations onto accuracy, response speed, and response repetition, separately and in interaction with the performed response (Go vs. NoGo). See Table S07 for inferential statistics from generalized additive models testing whether condition differences occurred selectively at particular time points within blocks. Incorrect responses were associated with significantly larger dilations compared to correct responses, an effect that was marginally stronger for NoGo responses (Fig. S09A). Over the time course of blocks, dilations were higher for incorrect NoGo responses than correct NoGo responses on cue repetitions 4 until 13, with no difference between incorrect and correct Go responses (Fig. S09D). Furthermore, slow responses were associated significantly with higher dilations compared to fast responses (Fig. S09B; note that on NoGo trials, no RTs can be observed) throughout blocks (Fig. S09E). Lastly, trials on which participants switched their response with respect to the last encounter of the same cue were associated with significantly higher pupil dilations (Fig. S09C) throughout a block (Fig. S09F), with no interaction with the performed response. In sum, incorrect responses, slower responses, and response switches were associated with stronger pupil dilations.

Both incorrect and slower responses were associated with significantly increased pupil dilations, but also with each other: incorrect responses (to NoGo cues) tended to be slower than correct responses (to Go cues; see Fig. 2E, F in the main text). We thus split trials with Go responses by both accuracy (correct/ incorrect) and response speed (fast/ slow; median split performed separately for correct and

incorrect responses for each participant) and tested whether both factors contributed independently to pupil dilations. Slower responses were associated with stronger dilations than faster responses irrespective of accuracy, while accuracy alone had no effect on dilations when controlling for response speed (Fig. S10 and inferential statistics in Tables S06 and S07). Hence, stronger pupil dilations on incorrect compared to correct responses follow from the former being slower than the latter. Note that GAMMs control for any changes in overall response speed or accuracy over time; the difference between fast and slow responses cannot be accounted for by increases in speed and accuracy over time.

Next, we investigated whether higher pupil dilations for Go responses to Avoid cues compared to Win cues were still observed for separate levels of accuracy, response speed (fast/ slow; median split performed separately for Win and Avoid cues for each participant), and response repetition. Dilations were still marginally significantly higher for response to Avoid cues than to Win cues irrespective of accuracy (Fig. S11A, Table S06). Additive models suggested significantly higher dilations for correct Go responses to Avoid than to Win cues on cue repetitions 4–13 as well as higher dilations for incorrect Go responses to Avoid than to Win cues on cue repetitions 6–16 (Fig. S11D, Table S07). Furthermore, while linear regression models suggested significantly higher dilations for slow than fast responses (median split performed separately for Win and Avoid cues), with no significant difference between Avoid and Win cues (Fig. S11B, Table S06), additive models suggested significantly higher dilations for slow responses to Avoid cues than slow responses to Win cues on cue repetitions 4–14, with no such difference for fast responses (Fig. S11E, Table S07). Lastly, while linear regression models indicated significantly higher dilations for response switches than response repetitions, with no differences between Avoid and Win cues (Fig. S11C, Table S06), additive models indicated that significantly higher dilations for response repetitions to Avoid than to Win cues on cue repetitions 3–13 (Fig. S11F, Table S07). For response switches, the pattern of differences was more complicated, with higher dilations for response switches for Avoid cues than for Win cues on the first three repetitions, but the reverse pattern on cue repetitions 6–13.

Taken together, these results suggest that dilations were indeed higher for Go responses to Avoid cues (for which participants had to overcome aversive inhibition) than Go responses to Win cues irrespective of accuracy, suggesting that the observed increase in pupil dilations cannot be attributed to

error processing. In fact, seemingly higher dilations to incorrect compared to correct responses are probably attributable to incorrect responses being relatively slower. Moreover, dilations were higher for Go responses to Avoid than to Win cues, but only for slow responses, with no such difference for fast responses. This pattern is in line with our interpretation of pupil dilation reflecting cognitive conflict and heightened physical effort recruitment in order to overcome aversive inhibition, a pattern that should lead to (and should only be observable on trials with) slow responses. In contrast, for fast responses, no such conflict might have occurred, potentially because these responses were made more “impulsively” and without proper processing of the cue or because responses had started to become well learned. Lastly, dilations on Go response repetitions (the large majority of responses) were higher for Avoid cues than Win cues, suggesting that this pattern was not induced by a different pattern of response switches for Avoid than Win cues. Notably, this pattern reversed for response switches. Note however that response switches towards Go were overall rare, and especially so for Win cues (i.e. the green dashed line in Fig. S11F reflects pupil dilations on those trials on which participants had previously performed a NoGo response to a Win cue and then decided to switch towards a Go response, likely because they deemed the previous response to be incorrect—a pattern that occurred very rarely in this task given that participants performed few NoGo responses to Win cues in the first place). In sum, these results are in line with our interpretation of heightened dilations for response to Avoid cues reflecting heightened physical effort recruitment in order to overcome aversive inhibition, a pattern associated with slow responses.

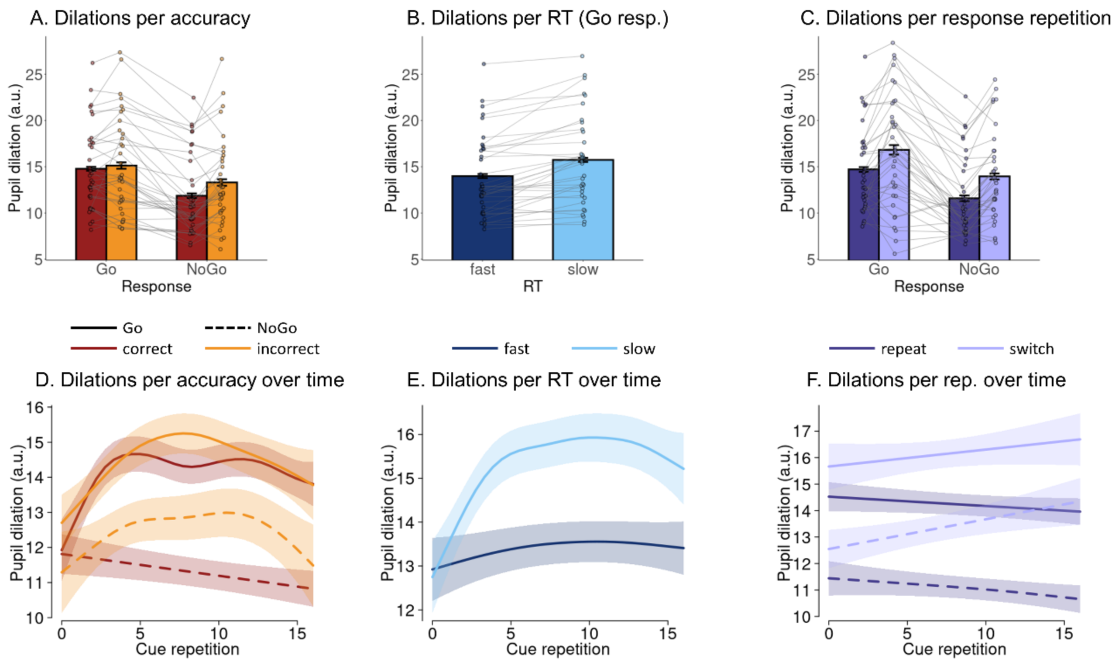

Figure S09. Association of pupil dilation with accuracy, response speed, and response repetition. **A.** Mean pupil dilation per response and accuracy (whiskers are ±SEM across participants, dots indicate individual participants). Dilations are significantly higher for Go than NoGo responses and higher for incorrect than correct responses (an effect that is marginally stronger for NoGo than Go responses). **B**. Mean pupil dilation per response speed (fast/ slow). Dilations are significantly higher for slow compared to fast responses. **C**. Mean pupil dilation per response and response repetition. Dilations are significantly higher for Go than NoGo responses and higher for response switches than response repetitions. **D**. Time course of dilations over cue repetitions (mean ± SE) as predicted from a generalized additive mixed-effects model (GAMM), separated by response and accuracy. Dilations are significantly stronger on trials with Go responses than on trials with NoGo responses throughout blocks. Furthermore, dilations are higher for incorrect than correct NoGo responses on repetitions 4–13. **E**. Time course of dilations over cue repetitions separated by response speed. Dilations are higher for slow compared to fast Go responses throughout blocks. **F**. Time course of dilations over cue repetitions separated by response and response repetition. Dilations are significantly stronger on trials with Go responses than on trials with NoGo responses and for response switches compared to response repetitions throughout blocks.

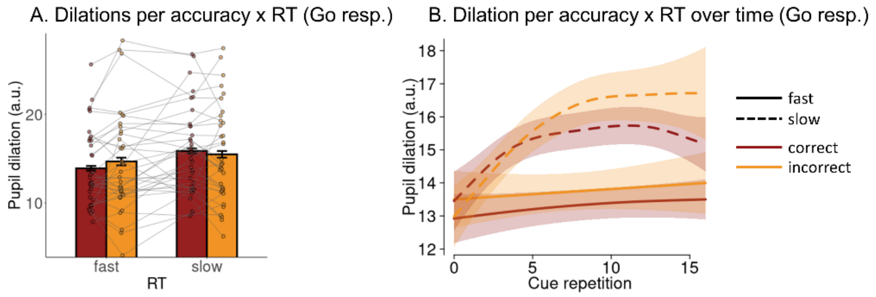

Figure S10. Association of pupil dilation with accuracy and response speed. **A.** Mean pupil dilation split by response speed and accuracy (whiskers are ±SEM across participants, dots indicate individual participants). Dilations are significantly higher on trials with slow responses than on trials with fast responses, with no significant differences between correct and incorrect responses. **B**. Time course of dilations over cue repetitions (mean ± SE) as predicted from a generalized additive mixed-effects model (GAMM), separated by accuracy and response speed. Dilations are significantly higher on trials with slow responses than on trials with fast responses, with no significant differences between correct and incorrect responses.

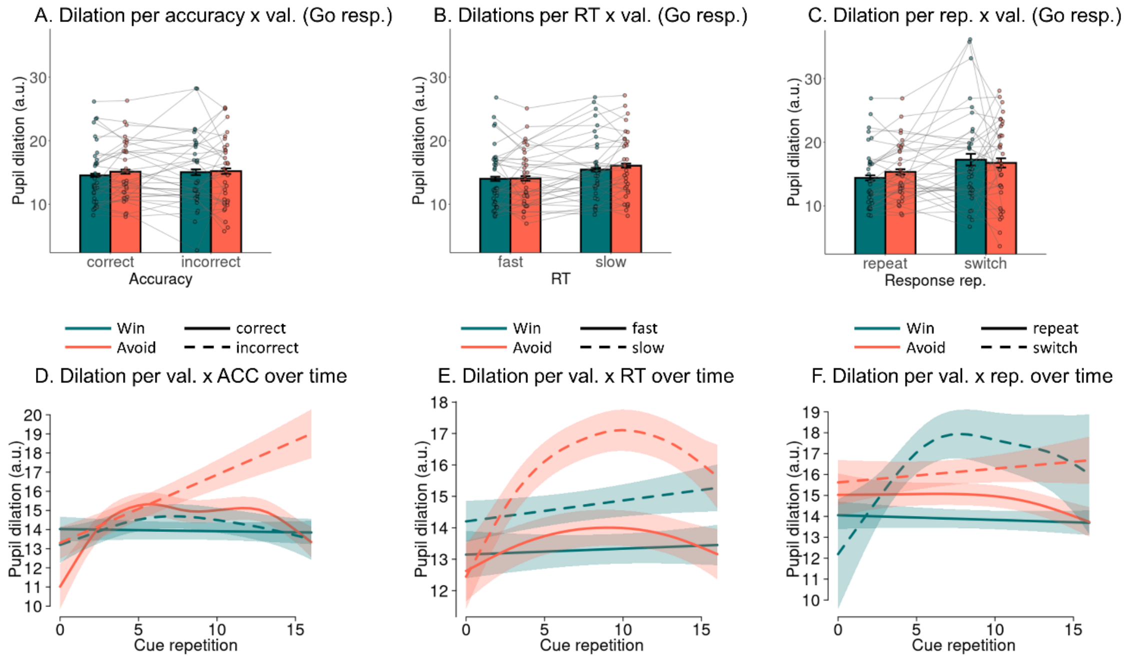

Figure S11. Higher pupil dilation for responses to Win compared to Avoid cues for trials split by accuracy, response speed, and response repetition. **A.** Mean pupil dilation on trials with Go responses per accuracy level per cue valence (whiskers are ±SEM across participants, dots indicate individual participants). Dilations are marginally significantly higher for responses to Avoid than to Win cues. **B**. Mean pupil dilation per response speed (fast/ slow) per cue valence. Dilations are significantly higher for slow compared to fast responses, while the effect of cue valence is not significant. **C**. Mean pupil dilation on trials with Go responses per response repetition per cue valence. Dilations are significantly higher for response repetitions to Avoid than to Win cues, while this effect is reversed for response switches. **D**. Time course of dilations over cue repetitions (mean ± SE) as predicted from a generalized additive mixed-effects model (GAMM), separated by accuracy and cue valence. Dilations are significantly stronger on for correct Go responses to Avoid than to Win cues on cue repetitions 4–13. Moreover, dilations are significantly stronger for incorrect Go responses to Avoid than to Win cues on cue repetitions 6–16. **E**. Time course of dilations over cue repetitions separated by response speed and cue valence. Dilations are significantly higher for slow compared to fast responses throughout blocks. Furthermore, dilations are significantly higher for slow responses to Avoid cues than to Win cues on cue repetitions 4–14, with no such difference for fast responses. **F**. Time course of dilations over cue repetitions separated by response repetition and cue valence. Dilations are significantly higher for response repetitions to Avoid than to Win cues on cue repetitions 3–13. Finally, dilations for response switches for Avoid cues are significantly higher than for Win cues on the first three repetitions, but this pattern reverses later, with stronger dilations for switches for Win cues than for Avoid cues on cue repetitions 6–13.

**Model ID**	**Trial subset**	**DV**	**IV**	***b***	***SE***	**$\chi^2(1)$**	***p***
1	All trials	Dilations	Accuracy (correct/ incorrect)	-0.039	0.012	8.267	.004
			Response (Go/ NoGo)	0.112	0.015	33.973	< .001
			Accuracy x Response	0.026	0.012	3.532	.060
2	Go responses	Dilations	RTs (fast/ slow)	-0.081	0.015	21.760	< .001
3	All trials	Dilations	Response repetition (repeat/ switch)	-0.105	0.019	22.924	< .001
			Response (Go/ NoGo)	0.139	0.019	34.249	< .001
			Response repetition x response	0.008	0.015	0.320	.571
4	Go responses	Dilations	Accuracy (correct/ incorrect)	-0.007	0.016	0.224	.636
			RTs (fast/ slow)	-0.073	0.017	14.429	< .001
			Accuracy x RTs	-0.018	0.016	1.386	.239
5	Go responses	Dilations	Accuracy (correct/ incorrect)	-0.017	0.017	1.099	.338
			Valence (Win/ Avoid)	-0.029	0.016	3.381	.071
			Accuracy x Valence	0.004	0.017	0.078	.730
6	Go responses	Dilations	RTs (fast/ slow)	-0.082	0.015	20.826	< .001
			Valence (Win/ Avoid)	-0.016	0.014	0.732	.392
			RTs x Valence	0.016	0.014	0.812	.368
7	Go responses	Dilations	Response repetition (repeat/ switch)	-0.107	0.027	12.841	< .001
			Valence (Win/ Avoid)	0.005	0.027	0.039	.844
			Response repetition x valence	-0.044	0.031	2.046	.153

Table S06. Results from mixed-effects linear regression models with trial-by-trial pupil dilation as dependent variable.

Model	**Parametric coefficient (Intercept difference)**	**Smooth (non-linear differences)**	**Windows of significant differences**
Accuracy *(all trials):*			
Go correct – Go incorrect	$t(5.870, 7.707) = 1.657, p = .098$	$F(1.001, 1.001) = 0.457, p = .499$	none
NoGo correct – NoGo incorrect	$t(4.460, 6.596) = 2.671, p = .008$	$F(3.573, 4.409) = 1.397, p = .198$	4 – 13
RTs *(Go responses):*			
Fast – slow	$t(5.710, 7.650) = 7.184, p < .001$	$F(1.422, 1.702) = 0.751, p = .364$	1 – 16
Repetition *(all trials):*			
Go repeat – Go switch	$t(6.054, 7.759) = 5.026, p < .001$	$F(1.000, 1.000) = 1.792, p = .181$	2 – 16
NoGo repeat – NoGo switch	$t(4.473, 6.606) = 5.904, p < .001$	$F(1.000, 1.000) = 1.823, p = .177$	1 – 16
Accuracy x RTs *(Go responses):*			
Slow Correct – Fast Correct	$t(5.107, 7.275) = 6.194, p < .001$	$F(1.000, 1.000) = 0.140, p = .709$	0 – 16
Slow Incorrect – Fast Incorrect	$t(3.000, 5.191) = 2.879, p = .004$	$F(1.000, 1.000) = 5.071, p = .025$	6 – 16
Fast Incorrect – Fast Correct	$t(3.970, 6.536) = 1.616, p = .106$	$F(1.003, 1.006) = 0.256, p = .617$	none
Slow Incorrect – Slow Correct	$t(6.416, 7.818) = 1.304, p = .192$	$F(1.000, 1.000) = 1.951, p = .163$	none
Accuracy x Valence *(Go responses):*			
Correct Avoid – correct Win	$t(5.182, 7.313) = 2.244, p = .025$	$F(4.479, 5.456) = 3.839, p = .001$	4 – 13
Incorrect Avoid – incorrect Win	$t(3.000, 5.253) = 2.159, p = .031$	$F(1.000, 1.000) = 2.573, p = .109$	6 – 16
RTs x Valence *(Go responses):*			
Fast Avoid – fast Win	$t(4.582, 6.825) = 0.958, p = .338$	$F(1.798, 2.176) = 0.408, p = .758$	none
Slow Avoid – slow Win	$t(5.974, 7.799) = 3.222, p = .001$	$F(2.384, 2.936) = 2.409, p = .065$	4 – 14
Repetition x Valence *(Go responses):*			
Repeat Avoid – repeat Win	$t(5.225, 7.400) = 3.246, p =.001$	$F(1.856, 2.278) = 0.869, p =.353$	3 – 13
Switch Avoid – switch Win	$t(5.710, 7.650) = 7.184, p < .001$	$F(1.422, 1.702) = 0.751, p =.364$	0 – 2, 6 – 13

Table S07. Results from generalized additive mixed models (GAMMs) with difference smooths between two conditions. The parametric term reflects a linear difference between conditions, while the smooth terms reflects any non-linear difference. Both add up to the total term. The time window of significant condition differences is automatically returned by the model. For the accuracy x RT and RT x valence models, the median split into fast and slow responses is performed separately for correct/ incorrect responses and Win/ Avoid cues for each participant.

Supplementary Material S08: Association of pupil baseline with accuracy, RTs, and response repetition over time

Beyond task-evoked trial-by-trial pupil dilations, past literature has also investigated pre-stimulus baseline pupil diameter as a potential readout of noradrenergic activity (Aston-Jones & Cohen, 2005; Eldar, Cohen, & Niv, 2013; Gilzenrat, Nieuwenhuis, Jepma, & Cohen, 2010). One the one hand, pupil baseline and task-evoked pupil dilation tend to be negatively correlated since high baseline leave less dynamic range for further dilations. In this sense, both measures could potentially capture similar phenomena and are partly redundant. However, on the other hand, pupil dilations are corrected for the immediately preceding pre-stimulus baseline and thus cannot reflect more "tonic" changes in pupil diameter on time scales longer than a single trial. In fact, pupil baseline itself tends to strongly decrease over the time course of an experiment (Muller, Mars, Behrens, & O'Reilly, 2019), likely reflecting decreases in arousal. These slower changes might reflect processes orthogonal to the trial-by-trial pupil dilations. Given that baselines are measured before cue onset, they cannot reflect the (randomized) task conditions (required action, valence, and arousal manipulation). Nonetheless, the process they reflect could still impact (or at least predict) task performance (responses, accuracy, and RTs).

While on the one hand, baseline pupil diameter could lead additional insights into cognitive processes beyond pupil dilation, on the other hand, caution is warranted given that possibility of spurious associations driven by time. When baseline pupil diameter decreases over time, any other variable that also changes on a similar time scale might be spuriously correlated with pupil diameter. Here, we used mixed-effects linear regression and generalized additive mixed effects models to test for effects of the baseline pupil diameter on responses, accuracy, and RTs (fast vs. slow, median split), controlling for potential linear and non-linear effects of time (cue repetition, 1–16).

See Table S08 for inferential statistics from mixed-effects linear regressions. See Fig. S12A-C for baselines per condition averaged over trials. When ignoring time, higher baseline pupil diameter was associated with a significantly higher propensity of Go responses, incorrect responses, and slower responses (see Table S08; Fig. S12A-C). The associations with accuracy and RTs disappeared when controlling for a linear effect of cue repetition (see Table S08). Most notably, additive models suggested

that baseline pupil diameter strongly decreased over time (Fig. S12D-F), with no significant difference between Go and NoGo responses, correct and incorrect responses, and only a minor (albeit significant) difference between fast and slow responses (Table S09; Fig. S12D-F) on the first eight cue repetitions, which was in fact of opposite sign (i.e., higher baselines before fast responses) to the results from the mixed-effect linear regression model (Fig. S12C). Thus, indeed, spurious associations between baseline pupil diameter and other variables arise through both changing over time, with participants showing less Go responses, less incorrect responses, and faster responses as they progress through a task block. In sum, there was strong evidence for baseline pupil diameter decreasing over the time course of a block, but no strong evidence for baseline pupil diameter affecting subsequent responses.

See Fig. S13A-C for the pupil dilation time course within a trial split by response and cue-valence when no baseline-correction is applied. Go responses to Avoid cues were associated with considerably stronger pupil dilations than Go responses to Win cues, However, this was partly driven by pre-existing baseline differences between those two trial types. Since baselines decreased with time, higher baselines on trials with Go responses to Avoid cues compared to those with Go responses to Win cues could potentially be explained by the former occurring relatively earlier within blocks (when baselines were still higher) than the latter. However, the opposite was the case: as participants learned the task, they showed more Go responses to Avoid cues with time, and the ratio between Go responses to Win and Avoid cues approached 50:50 with time. Hence, the overall decay in baseline cannot explain baseline differences between these two trial types. In fact, baseline differences were even stronger in the second half of blocks (Fig. S13C) compared to the first half (Fig. 13B), i.e. they prevailed and became even stronger as the ratio of both trial types approached 50:50. A generalized additive model corroborated that pupil baselines were significantly higher on trials with Go responses to Avoid cues compared to trials with Gon responses to Win cues in the second half of blocks (Fig. S13D, E; Table S09). In sum, Go responses to Avoid cues were not only associated with higher pupil dilations, but also higher pupil baselines, suggesting that pre-existing differences arousal before cue onset might have contributed to the mobilization of effort and invigoration of Go responses against aversive Pavlovian biases.

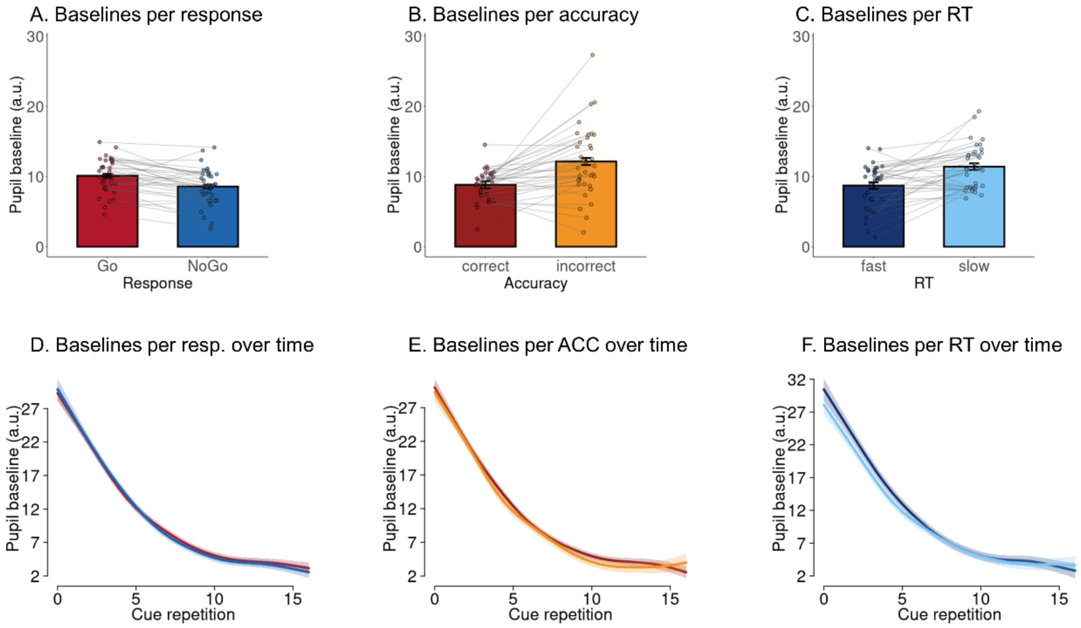

Figure S12. Relationship of pre-trial baseline pupil diameter with responses, accuracy, and RTs. **A.** Pupil pre-trial baseline split by the response made on the trial (whiskers are ± SEM across participants, dots indicate individual participants). Considering trials irrespective of their temporal position within a block, baseline pupil diameter is significantly higher before trials with Go responses than trials with NoGo responses. **B**. Pupil baseline split by the speed of the response made on the following trial (only trials with Go responses). Considering trials irrespective of their temporal position within a block, baseline pupil diameter is significantly higher before trials with incorrect responses than trials with correct responses. **C**. Pupil baseline split by the accuracy of the response made on the following trial. Considering trials irrespective of their temporal position within a block, baseline pupil diameter is significantly higher before trials with slow responses than trials with fast responses. **D**. Time course of baseline pupil diameter over cue repetitions (mean ± SE) as predicted by a generalized additive mixed-effects model (GAMM), separated by responses. There is no significant difference between trials with Go and NoGo responses. **E.** Time course of baseline pupil diameter over cue repetitions as predicted by a generalized additive mixed-effects model (GAMM), separated by accuracy. There is no significant difference between trials with Go and NoGo responses. **F.** Time course of baseline pupil diameter over cue repetitions (mean ± SE) as predicted by a generalized additive mixed-effects model (GAMM), separated by response speed (fast/ slow; median split). For the first eight cue repetitions, baseline pupil diameter is higher before fast compared to slow responses.

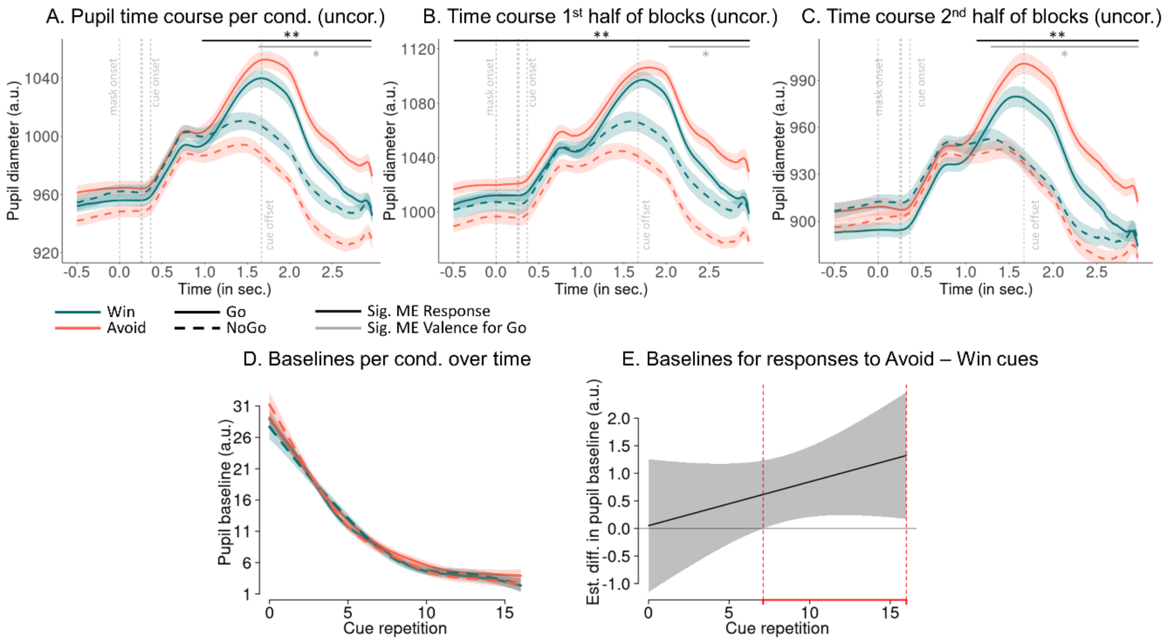

Figure S13. Pupil time course within a trial per response per cue valence without baseline correction (mean ± SEM across participants). **A.** Pupil time course split by cue valence and response made (whiskers are ± SEM across participants, dots indicate individual participants). Vertical dashed lines indicate the onset of the forward mask (at 0 ms), the prime (at 250 ms), the backwards mask (at 266 ms), the cue onset (at 366 ms), and the cue offset (at 1666 ms). The pupil dilates significantly more strongly on trials with Go responses than on trials with NoGo responses (cluster above threshold: 917–2,966 ms; *p* < .001; longer black horizontal line). Furthermore, within this time window, the pupil dilates significantly more strongly and sustainedly for responses to Avoid than to Win cues (cluster above threshold: 1,545–2,966 ms; *p* = .011; shorter black horizontal line). Note however that pre-cue pupil baselines are already higher for Go responses to Avoid cues than Go responses to Win cues. **B.** When repeating this analysis for only the first half of trials within a block, the pupil is wider on trials with Go responses than on trials with NoGo responses throughout the entire time window (cluster above threshold: -1,000–2,966 ms; *p* < .001; longer black horizontal line) and, within this time window, wider for Go responses to Avoid than to Win cues (cluster above threshold: 2,038–2,966 ms; *p* = .049; short black horizontal line). **(C)** In the second half of trials, the pupil is wider on trials with Go responses than on trials with NoGo responses in a more restricted time window (cluster above threshold: 1,137–2,966 ms; *p* < .001) and, within this time window, wider for Go responses to Avoid than to Win cues (cluster above threshold: 1,262–2,966 ms; *p* < .001). The fact that the differences in pupil diameter for Go responses to Avoid cues compared to responses to Win cues gets larger with time suggests that people learn to mobilize effort to invigorate Go responses against the Pavlovian bias (aversive inhibition) present on trials with Avoid cues. **D.** Time course of pupil baselines over cue repetitions (mean ± SE) as predicted from a generalized additive mixed-effects model (GAMM), separated by response and cue valence. Baselines are significantly stronger on trials with Go responses than on trials with Go responses to Avoid cues than trials with Go responses to Win cues from cue repetition 7 to 16, putatively reflecting that pre-cue fluctuations in arousal contribute to the invigoration of Go response against aversive Pavlovian biases. **E.** Difference line between baselines on trials with responses to Avoid cues minus Win cues. Areas highlighted in red indicate time windows with significant differences.

Model ID	DV	IV	*b*	*SE*	$\chi^2(1)$	*p*
1	Pupil baseline	Response (Go/ NoGo)	0.048	0.012	13.961	< .001
2	Pupil baseline	Accuracy (correct/ incorrect)	-0.103	0.021	18.692	< .001
3	Pupil baseline	RTs (fast/ slow)	-0.082	0.020	13.906	< .001
4	Pupil baseline	Response (Go/ NoGo)	0.023	0.010	5.336	.021
		Cue repetition (1–16, z-scored)	-0.399	0.032	60.144	< .001
		Response x cue repetition	-0.026	0.010	6.814	.009
5	Pupil baseline	Accuracy (correct/ incorrect)	0.025	0.015	2.799	.094
		Cue repetition (1–16, z-scored)	-0.429	0.034	60.685	< .001
		Accuracy x cue repetition	0.039	0.012	9.396	.002
6	Pupil baseline	RTs (fast/ slow)	0.015	0.013	0.806	.369
		Cue repetition (1–16, z-scored)	-0.422	0.033	8.646	.003
		RTs x cue repetition	0.009	0.020	0.137	.711

Table S08. Results from mixed-effects linear regression models with trial-by-trial baseline pupil diameter as dependent variable.

Model	**Parametric coefficient (Intercept difference)**	**Smooth (non-linear differences)**	**Windows of significant differences**
***Response**:*			
Go – NoGo	*t*(4.777, 9.307) = -1.052, *p* =.293	*F*(1.000, 1.001) = 0.261, *p* =.616	none
***Accuracy**:*			
Correct – incorrect	*t*(4.798, 9.296) = -1.867, *p* =.062	*F*(1.129, 1.240) = 0.381, *p* = 0.73	none
RTs:			
Fast – slow	*t*(4.584, 8.867) = -1.809, *p* =.071	*F*(1.000, 1.000) = 4.700, *p* =.030	0 – 8
Cue valence (Go responses):			
Avoid – Win	*t*(4.423, 8.725) = 2.350, *p* = .019	*F*(1.000, 1.000) = 1.385, *p* =.239	7 – 16

Table S09. Results from generalized additive mixed models (GAMMs) with difference smooths between two conditions. The parametric term reflects a linear difference between conditions, while the smooth terms reflects any non-linear difference. Both add up to the total term. The time window of significant condition differences is automatically returned by the model.

Supplementary Material S09: Outcome-locked pupil dilation

Apart from cue- (or masked-) locked pupil dilation, we also investigated outcome-locked pupil dilation (epoched from -1000 ms before until 2000 ms after outcome onset) as a function of the obtained outcome and the previously made response.

See Table S10 and Fig. S14 for results from mixed-effects linear regression models as well as post-hoc z-tests contrasting conditions against each other. Pupil dilations were significantly stronger on trials with punishments compared to trials with rewards or neutral outcomes, while trials with rewards and neutral outcomes were not significantly different from each other. Dilations were not different between trials on which neutral outcomes signaled the absence of rewards compared to trials on which they signaled the absence of punishments.

When analyzing dilations as a function of both the obtained outcome and the previously made response, we observed main effects of outcome and response, while the interaction between them was not significant (Table S10). Pupil dilations were higher after NoGo responses compared to Go responses (Fig. S15A). However, inspection of the raw pupil time course within a trial revealed that this difference was an artifact of baseline correction: raw pupil time courses tended to be higher after Go compared to NoGo responses (for trials with punishment and neutral outcomes; Fig. S15C), leaving less dynamic range for further increases on Go compared to NoGo trials and thus leading to lower (baseline-corrected) pupil dilations on Go compared to NoGo trials (Fig. S15B).

In sum, the pupil dilated more strongly in response to punishments compared to rewards or neutral outcomes.

Model ID	DV	IV	$\chi^2(1)$	z	p
1	Pupil dilation	Outcome valence (positive/ negative)	13.439		< .001
2	Pupil dilation	Outcome displayed (reward/ neutral/ punishment)	27.237		< .001
		Punishment – neutral		6.351	< .001
		Punishment – reward		5.473	< .001
		Neutral – reward		1.093	.519
3	Pupil dilation	Outcome interpreted (rew./ no rew./ no pun./ pun.)	31.251		< .001
		Punished vs. not punished		5.591	< .001
		Punished vs. not rewarded		6.996	< .001
		Punished vs. rewarded		5.457	< .001
		Not punished vs. not rewarded		1.586	.387
		Not punished vs. rewarded		0.321	.989
		Not rewarded vs. rewarded		2.021	.180
4	Pupil dilation	Outcome displayed (reward/ neutral/ punishment)	25.704		< .001
		Response (Go/ NoGo)	19.116		< .001
		Outcome displayed x response	1.306		.521

Table S10. Results from mixed-effects linear regression models with outcome-locked trial-by-trial pupil dilation as dependent variable. Differences between any conditions were first tested with χ^2 tests and then followed up with z-tests testing two conditions against each other. *P*-values for the follow-up z-tests are corrected for multiple comparisons using the Tukey method.

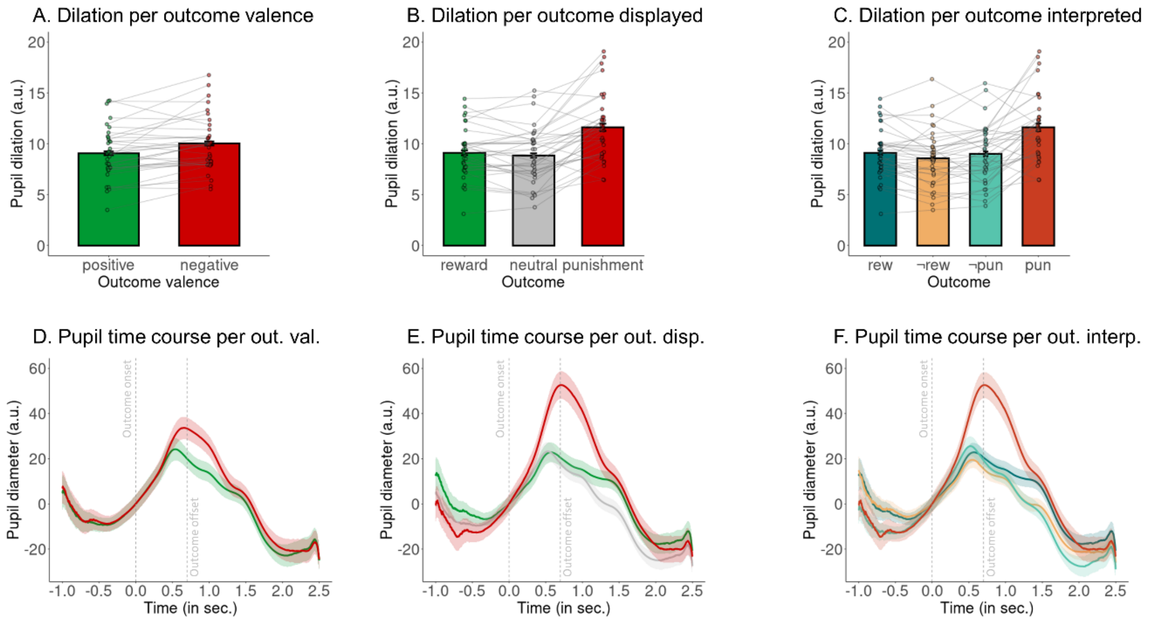

Figure S14. Effect of outcomes on outcome-locked pupil dilation. Pupil dilation as a function of outcome valence (**A**), the displayed outcome (**B**) or the outcome interpreted (with neutral outcomes recognized as signaling the absence of a reward/ punishment, **C**; whiskers are ± SEM across participants, dots indicate individual participants). The pupil dilates more strongly on trials with punishments compared to rewards or neutral outcomes. (**D-F**) Pupil time course within a trial separately for the different outcome conditions (mean ± SEM across participants; baseline-corrected). Vertical dashed line represent the onset (at 0 ms) and offset (at 700 ms) of outcomes.

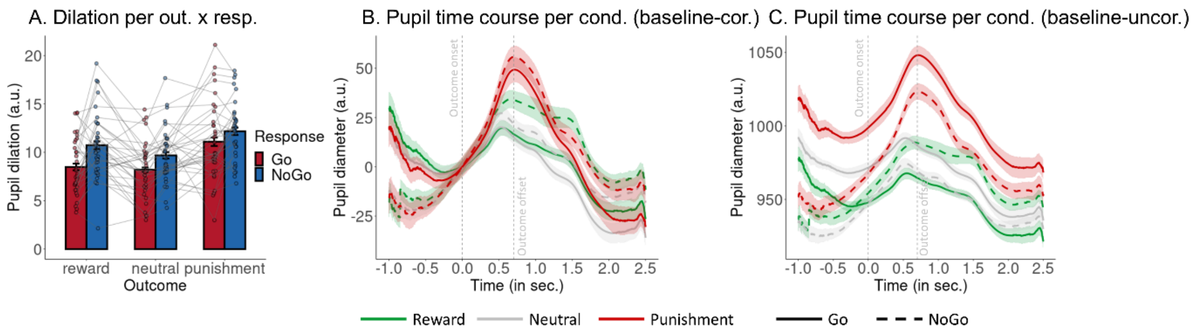

Figure S15. Effect of outcomes and responses on outcome-locked pupil dilation. **A.** Pupil dilation as a function of outcome displayed and the response performed on the same trial manipulation (whiskers are ±SEM across participants, dots indicate individual participants). When applying baseline-correction for differences in the time window of 500 ms before outcome onset, dilations are significantly higher on trials with punishments compared to trials with rewards or neutral outcomes and higher on trials with NoGo than trials with Go responses. **B**. Pupil time course within a trial separately per outcome and response condition (mean ± SEM across participants; baseline-corrected). It appears that for trials with rewards and neutral outcomes, pupil dilations are higher after NoGo than Go responses. Vertical dashed line represent the onset (at 0 ms) and offset (at 700 ms) of outcomes. **C**. Same as panel B, but not baseline corrected. It becomes clear that the pupil time course is higher after Go compared to NoGo responses, leaving less room for further increase on trials with Go compared to NoGo responses, explaining while the baseline-corrected dilations tends to be smaller after Go than NoGo responses.

Supplementary References

Allen, M., Frank, D., Schwarzkopf, D. S., Fardo, F., Winston, J. S., Hauser, T. U., & Rees, G. (2016). Unexpected arousal modulates the influence of sensory noise on confidence. *eLife*, *5*, 1–17. doi: 10.7554/eLife.18103

Aston-Jones, G., & Cohen, J. D. (2005). An integrative theory of locus coeruleus-norepinephrine function: Adaptive gain and optimal performance. *Annual Review of Neuroscience*, *28*, 403–450. doi: 10.1146/annurev.neuro.28.061604.135709

Cyders, M. A., Littlefield, A. K., Coffey, S., & Karyadi, K. A. (2014). Examination of a short English version of the UPPS-P Impulsive Behavior Scale. *Addictive Behaviors*, *39*(9), 1372–1376. doi: 10.1016/j.addbeh.2014.02.013

Eldar, E., Cohen, J. D., & Niv, Y. (2013). The effects of neural gain on attention and learning. *Nature Neuroscience*, *16*(8), 1146–1153. doi: 10.1038/nn.3428

Gilzenrat, M. S., Nieuwenhuis, S., Jepma, M., & Cohen, J. D. (2010). Pupil diameter tracks changes in control state predicted by the adaptive gain theory of locus coeruleus function. *Cognitive, Affective & Behavioral Neuroscience*, *10*(2), 252–269. doi: 10.3758/CABN.10.2.252

Mudrik, L., & Deouell, L. Y. (2022). Neuroscientific evidence for processing without awareness. *Annual Review of Neuroscience*, *45*(1), 403–423. doi: 10.1146/annurev-neuro-110920-033151

Muller, T. H., Mars, R. B., Behrens, T. E., & O'Reilly, J. X. (2019). Control of entropy in neural models of environmental state. *eLife*, *8*, 1–30. doi: 10.7554/eLife.39404

Skora, L. I., Livermore, J. J. A., Dienes, Z., Seth, A. K., & Scott, R. B. (2023). Feasibility of unconscious instrumental conditioning: A registered replication. *Cortex*, *159*, 101–117. doi: 10.1016/j.cortex.2022.12.003

Spielberger, C., Gorssuch, R., Lushene, P., Vagg, P., & Jacobs, G. (1983). *Manual for the State-Trait Anxiety Inventory.* Moutain View, CA: Consulting Psychologists Press.

Vadillo, M. A., Malejka, S., Lee, D. Y. H., Dienes, Z., & Shanks, D. R. (2022). Raising awareness about measurement error in research on unconscious mental processes. *Psychonomic Bulletin & Review*, *29*(1), 21–43. doi: 10.3758/s13423-021-01923-y
